# The endophyte *Fusarium oxysporum* 3RR genome analysis identifies bioactive compounds targeting biofilm formation

**DOI:** 10.3389/fmicb.2026.1841716

**Published:** 2026-09-21

**Authors:** Syrine Nebli, Oumaima Saadaoui, Héla Gargouri, Cyrine Abid, Marwa Jardak, Fida Djebali, Salma Loukil, Riadh Ben Marzoug, Lobna Jlaiel, Séverine Croze, Joel Lachuer, Najla Kharrat, Sami Mnif

**Affiliations:** 1 Laboratory of Molecular and Cellular Screening Processes, Centre of Biotechnology of Sfax, University of Sfax, Sfax, Tunisia; 2 Analytical Service Unit, Center of Biotechnology of Sfax (CBS), University of Sfax, Sfax, Tunisia; 3 Université Claude Bernard Lyon 1 (UCBL), Plateforme ProfileXpert de Génomique et Microgénomique, SFR Santé-Lyon-Est, CNRS UMR-S3453, INSERM US7, Faculté de Pharmacie de Lyon, University of Lyon, Lyon, France; 4 Université Claude Bernard Lyon 1 (UCBL), Plateforme ProfileXpert de Génomique et Microgénomique, SFR Santé-Lyon-Est, CNRS UMR-S3453, INSERM US7, Faculté de Pharmacie de Lyon, University of Lyon, Lyon, France; 5 Université Claude Bernard Lyon 1 (UCBL), UMR INSERM 1052, CNRS 5286, Centre Léon Bérard, Centre de Recherche en Cancérologie de Lyon (CRCL), University of Lyon, Lyon, France

**Keywords:** antibacterial activity, biofilm inhibition, endophytic fungi, *Fusarium oxysporum*, genome sequencing, secondary metabolites

## Abstract

**Introduction:**

The rising global challenge of antimicrobial resistance and biofilm-associated infections necessitates the discovery of novel bioactive compounds. Endophytic fungi, recognized for their metabolic versatility and ecological adaptability, represent a promising, yet underexplored, source of such molecules. This study aimed to comprehensively characterize the biosynthetic potential of *Fusarium oxysporum* strain 3RR and establish mechanistic links between its genomic architecture, enzymatic capacity, and observed antibiofilm and antibacterial activities.

**Methods:**

An integrated genomics-metabolomics approach was employed. Whole-genome sequencing combined Illumina paired-end and Oxford Nanopore long-read technologies, with hybrid assembly via SPAdes. Gene prediction was performed using BRAKER3 followed by functional annotation via InterProScan, eggNOG-mapper, while biosynthetic gene clusters (BGCs) were predicted using antiSMASH v8.0.4. Antibacterial activity was assessed by agar disk diffusion and microdilution, and antibiofilm activity by crystal violet quantification and microscopy. The extract composition was analyzed using Gas Chromatography–Mass Spectrometry (GC-MS).

**Results:**

The assembled genome (41.14 Mb, N50 = 3.52 Mb) was annotated, and predicted to contain 11,738 genes. 36 BGCs were identified, predominantly terpenes, NRPS, and PKS clusters. CAZy analysis revealed a notable CE10 esterase expansion. GC-MS identified 14 major compounds in the extracellular ethyl acetate extract, dominated by 2,4-di-tert-butylphenol (2,4-DTBP, 16.72%), oleic acid (10.14%), and 8-hydroxyisotrichodermin (7.79%). The extract exhibited antibacterial activity against *Staphylococcus epidermidis* (up to 34 mm inhibition zone) and *Escherichia coli*, and remarkable antibiofilm effects, inhibiting *S. epidermidis* biofilm formation by 96.11% and eradicating *Staphylococcus aureus* biofilms by up to 54.85%, confirmed microscopically.

**Discussion:**

The study reveals a sophisticated metabolic architecture in *F. oxysporum* 3RR, driven by CAZy enzymes and secondary metabolite BGCs. CE10 may act as a metabolic interface by facilitating the liberation or transformation of phenolic and lipid-derived compounds, thereby increasing their potential bioavailability. Rather than attributing the observed bioactivity to 2,4-DTBP or oleic acid alone, the present work places these known metabolites within their genomic and ecological context, linking them to the strain’s biosynthetic capacity and its plant-endophytic lifestyle.

**Conclusion:**

*F. oxysporum* 3RR possesses a powerful chemical defense and offense system, positioning this endophytic fungus as a promising source for novel therapeutic agents against microbial biofilms.

## Introduction

Endophytic fungi are microorganisms that live within the internal tissues of plants without causing visible disease symptoms, at least for a portion of their life cycle (Morelli et al., 2020; Baron and Rigobelo, 2021). These symbiotic microorganisms are vastly distributed across diverse plants and ecological systems and play significant roles in plant growth, stress tolerance, and ecological adaptation (Wen et al., 2022). Endophytes can enhance host fitness by producing phytohormones, improving nutrient acquisition, and synthesizing bioactive compounds that protect plants against pathogens, herbivores, and environmental stresses (Akram et al., 2023; Baron and Rigobelo, 2021). Due to their remarkable metabolic versatility and ecological importance, endophytic fungi have attracted considerable attention as a promising source of novel natural products with applications in agriculture, medicine, and biotechnology (Wen et al., 2022; Zhang et al., 2022b). Advances in genomics and metabolomics have further revealed the immense diversity of endophytic fungi and their biosynthetic potential, highlighting their role as an untapped reservoir of valuable bioactive compounds (Turco et al., 2023; Patra et al., 2025; Kharte et al., 2025).

In parallel, microbial biofilms, organized communities of microorganisms enclosed within a self-produced extracellular matrix, have emerged as a major factor in chronic infections, antimicrobial resistance, and industrial biofouling (Hall-Stoodley et al., 2004). Biofilm-associated resistance mechanisms limit the efficacy of conventional antibiotics, stressing the emergency for novel strategies to inhibit biofilm formation or disrupt established biofilms (Rather et al., 2021). In this context, endophytic microorganisms, including fungi and bacteria, have shown increasing interest as sources of biofilm-inhibitory and quorum-sensing-modulating molecules (Abdel Razek et al., 2020; Singh and Dubey, 2020; Sharma et al., 2024), reflecting their adaptation to complex plant-microbe interactions and their capacity to produce structurally diverse bioactive metabolites (Gilmore et al., 2022; Iungin et al., 2024). In this context, targeting biofilm regulatory pathways rather than directly inhibiting bacterial growth has emerged as a promising strategy to reduce selective pressure associated with antimicrobial resistance development (Zhu et al., 2026).

Among endophytic fungi, species of the genus *Fusarium* are particularly widespread and display a broad ecological versatility, acting as saprophytes, endophytes, or plant pathogens depending on the strain and environment (Morelli et al., 2020). *Fusarium oxysporum* (ncbitaxon:5507), a species complex commonly found in soils and plant-associated habitats, includes strains that colonize host plants asymptomatically as endophytes as well as pathogenic variants responsible for severe vascular wilts in numerous crops (Gordon and Martyn, 1997; Edel-Hermann and Lecomte, 2019; Wang et al., 2022; Jackson et al., 2024). Endophytic strains of *F. oxysporum* have received increasing attention for their ability to interact with host plants and produce a rich array of secondary metabolites whose structures and biological roles underpin both ecological adaptation and pharmacological potential (de Lamo and Takken, 2020; Ibrahim et al., 2021). These metabolites contribute to a remarkable spectrum of bioactivities, including antimicrobial, antiviral, antioxidant, insecticidal, and anticancer effects, making endophytic *Fusarium* species a promising reservoir of novel compounds for pharmaceutical applications (Manganyi and Ateba, 2020; Wen et al., 2022; Kubayi et al., 2025).

In recent years, genome sequencing and bioinformatic tools have enabled recognition of gene clusters involved in the biosynthesis of these compounds, facilitating the discovery of new natural products and the exploration of fungal metabolic capabilities (Medema et al., 2015; Robey et al., 2021). In particular, AntiSMASH-based analyses have revealed that many fungal genomes contain a high proportion of orphan biosynthetic gene clusters with no similarity to known pathways, emphasizing the hidden metabolic diversity of endophytic fungi (Zhu et al., 2026). Consequently, studying the genomic and metabolic potential of endophytic fungi such as *F. oxysporum* represents a promising strategy for uncovering novel bioactive compounds and advancing sustainable biotechnological applications (Toghueo, 2019; Manganyi and Ateba, 2020; Ibrahim et al., 2021).

To comprehensively characterize the biosynthetic potential of *F. oxysporum* endophytic strains, integrated approaches combining genome sequencing, biosynthetic gene cluster (BGC) identification, and metabolite profiling are increasingly used. These strategies help establish links between genomic features and secondary metabolite production (Hautbergue et al., 2018).

In this study, we employed an integrated genomic–metabolomic approach to investigate the secondary metabolite potential of an endophytic *F. oxysporum* strain isolated from *Juniperus oxycedrus*. To the best of our knowledge, this represents the first genome sequencing study of an endophytic *F. oxysporum* strain recovered from this host plant (Makhloufi et al., 2021). By linking metabolite production with genomic architecture, this work provides a functional framework for understanding the biosynthetic and ecological potential of plant-associated non-pathogenic members of the *Fusarium oxysporum* species complex.

The overall objective of this work is to comprehensively characterize the biosynthetic potential of the endophytic fungus *Fusarium oxysporum*, and to identify and prioritize secondary metabolites with potential antibiofilm and anti-quorum-sensing activities. By linking biosynthetic gene cluster architecture and metabolite production, our study aims to contribute to the development of innovative strategies to combat biofilm-associated microbial resistance and to explore endophytic-derived molecules as promising tools for biofilm control and microbial communication interference.

## Materials and methods

### Isolation of the endophytic fungi 3RR

Leaf surfaces of *Juniperus oxycedrus* were subjected to a surface sterilization protocol prior to endophyte isolation (Kandel et al., 2017; Deepak and Virk, 2022). Sterilized leaf fragments were aseptically cut into small pieces using a sterile scalpel and placed (4–5 per tube) in sterile 2-ml Eppendorf tube with 700 μL Potato Dextrose Broth (PDB, see composition in Supplementary Table S1). Tubes are incubated at 30 °C for 7–10 days until visible fungal growth appears on the culture medium. Visible growth is transferred *via* platinum loop to Potato Dextrose Agar (PDA, composition in Supplementary Table S1) petri dishes and incubated at 30 °C. Individual hyphal tips are isolated onto 10-cm PDA plates. Leaf fragments are cultured up to 1 month. Isolated endophytic strains are deposited in the laboratory of molecular and cellular screening processes (Center of Biotechnology of Sfax, Tunisia) collection and maintained in triplicate (1 mL glycerol:water, 1:1) at −80 °C in 2-ml tubes.

### Genomic DNA extraction and ITS amplification

Genomic DNA (gDNA) was isolated from growing 3RR strain mycelium on solid PDA using DNAeasy PlantPro Kit (Qiagen) according to manufacturer instructions. Quality, quantity and gDNA integrity were then assessed by NanoDrop^®^-2000 instrument (Thermo Scientific, United States) and fluorometer (Qubit 2.0) (Invitrogen) using Invitrogen QubitTM dsDNA BR Assay Kit (ThermoFisher Scientific), according to the manufacturer instructions. Final quality control was performed by High Sensitivity DNA Assay kit (Agilent Technologies) on Agilent 2,100 Bioanalyzer.

ITS (including ITS1, 5.8 S and ITS2) regions of the rDNA were amplified from gDNA samples using the polymerase chain reaction and primer sets ITS1 (5′-TCCGTAGGTGAACCTGCG G- 3′)/ITS4 (5′-TCCTCCGCTTATTGATATGC- 3′) (White et al., 1990). The amplified regions were thus sequencing with SANGER sequencing. The related sequences (forward and reverse) were collected and aligned using the CLUSTALW software (Larkin et al., 2007), analyzed using BLAST^1^ (Altschul et al., 1990, 1997). The phylogenetic tree was constructed using the neighbor-joining method (Saitou and Nei, 1987) with MEGA software (Tamura et al., 2007). The topology of the distance tree was tested by resampling data with 1,000 bootstraps to provide confidence estimates.

The data generated in this study (3RR BioSample, SRA for P2Solo raw long-reads, SRA for Illumina raw short-reads and ITS GenBank accession numbers) have been deposited in the BioProject “FOWGS BIOFILM” under the accession number PRJNA1444486 (http://www.ncbi.nlm.nih.gov/bioproject/1444486).

### Library preparation for sequencing technologies

The Illumina library was generated by Illumina DNA Prep Kit (Illumina Technology, San Diego), according to the standard Illumina protocol, and sequenced on NextSeq550 with mid output in paired-end mode with 2 × 150 bp read lengths.

Rapid Barcoding Sequencing Kit V14 (SQK-RBK114.24) was employed for ONT library preparation following the manufacturer’s instruction. The raw data was obtained after 72 h running time on PromethION 2 Solo with flow cell R10.4.1 using a predefined base calling model.

### Genome assembly

All scripts and reproducible notebooks used for genome assembly, annotation, and comparative genomics are publicly available at: https://github.com/SyrineNebli/fusarium-3RR-genome.

#### Preliminary data analysis

##### Sequencing data

Whole-genome sequencing data for *Fusarium oxysporum* was performed using a hybrid approach combining two complementary sequencing technologies: Illumina paired-end sequencing, which provides high base-level accuracy but is limited in resolving repetitive regions and structural complexity, and Oxford Nanopore long-read sequencing, which overcomes these limitations by generating reads spanning several kilobases, enabling the resolution of repetitive elements and achieving greater assembly contiguity.

The Illumina dataset comprised 10.6 million Illumina paired-end reads (35–149 bp), totaling 1.5 Gb of sequence data with a GC content of 47%, while Nanopore sequencing yielded 2,463,762 reads totaling 3.66 Gb, with an N50 read length of 2,484 bp.

##### Quality control

Initial quality assessment of Illumina reads was performed using FastQC (v0.12.1, https://github.com/s-andrews/FastQC), while Nanopore reads were evaluated using NanoPlot (v1.42.0) (De Coster and Rademakers, 2023). These analyses assessed base quality scores, read length distribution, GC content, and potential adapter contamination.

##### Read preprocessing

Illumina reads were filtered and trimmed using fastp (v1.0.1) (Chen et al., 2018) to remove adapter sequences, low-quality bases, and poly-G/poly-X tails, retaining reads with an average quality ≥28 and minimum length ≥101 bp. Overlapping read pairs were merged to generate longer consensus reads while preserving unmerged paired reads. Nanopore reads also were filtered using fastp, retaining reads with average quality ≥16 and minimum length ≥1,000 bp.

##### K-mer analysis

K-mer analysis was performed on Illumina sequencing data using KAT (v2.4.2) (Mapleson et al., 2017) across a range of k-mer sizes (21–101), generating histograms and genome size estimates from all read types (R1, R2, merged) to assess genome characteristics, heterozygosity, and repetitive content.

#### Hybrid genome assembly

Hybrid assembly was performed with SPAdes (v4.2.0) (Bankevich et al., 2012), combining Illumina paired-end, merged reads, and Nanopore long reads. Assemblies used multiple k-mer sizes (25–127) and isolate mode for fungal genome optimization. Assembly quality was evaluated using total assembly size, N50, largest contig, and number of contigs, with further assessment planned via QUAST (v5.3.0) (Gurevich et al., 2013).

To improve contiguity of the hybrid assembly, we performed secondary scaffolding using Ragout (v2.3) (Kolmogorov et al., 2014) with multiple reference genomes, as the initial assembly remained fragmented and limited chromosome-level analyses.

Seven high-quality *Fusarium oxysporum* genomes were downloaded from NCBI: Fo47, ZUM2407, V032g, ME23, LD-06, Fo5176, and Race 4. All FASTA files were decompressed and standardized.

Genome distances were calculated with Mash (v2.3) (Ondov et al., 2016), and a UPGMA tree was built in Python (v3.10.12) in strictly binary Newick format for Cactus compatibility.

Cactus (v2.9.9) (Armstrong et al., 2020) generated a HAL alignment, converted to MAF format, which served as input for Ragout to order and orient contigs, producing longer scaffolds. This latter was evaluated with QUAST for contiguity. The completeness and quality of the assembled genome were assessed using BUSCO (Benchmarking Universal Single-Copy Orthologs, v6.0.0) with the Ascomycota_odb12 dataset (Seppey et al., 2019).

To evaluate the taxonomic origin of the assembled genome and detect potential contamination, taxonomic classification was performed using Kraken2 (v2.1.6). The classification was performed against a comprehensive Kraken2 reference database containing genomes from bacteria, archaea, viruses, fungi, and other eukaryotes (Wood et al., 2019).

### Functional annotation

Repetitive elements were identified and masked prior to gene prediction to improve annotation accuracy. Repeat analysis was performed using RepeatMasker v4.2.0 within the Dfam tetools container (tetools_lib) (Tempel, 2012), using a combined repeat library consisting of curated fungal repeats from the Dfam database (release 3.9, partition 16) and *de novo* repeat families identified with RepeatModeler (Flynn et al., 2020). This integrated approach enabled the detection of both known and species-specific transposable elements in the assembled genome.

The masked genome assembly of *F. oxysporum* was used as reference. A custom fungal protein database was created from OrthoDB (odb12v2) using selectClade.py to extract Fungi sequences. The resulting FASTA was used as homology evidence for annotation.

Gene prediction was performed with BRAKER3 (Gabriel et al., 2024), which combines GeneMark-EP (v 4.72) (Brůna et al., 2020) and AUGUSTUS (v 3.5.0) (Hoff and Stanke, 2019). Annotation used protein evidence only, training a new species model with the fungal branch point model and multithreading.

Predicted protein sequences generated by BRAKER3 were used in the functional annotation. Protein sequences were scanned using InterProScan (v5.75–106.0) (Jones et al., 2014) and eggNOG-mapper (v2.1.13) (Cantalapiedra et al., 2021), with a custom Fungi-specific database built from eggNOG DB v5.0.2. The mapping used DIAMOND v2.1.12 (Buchfink et al., 2015) for sequence alignment and MMseqs2 v17.b804f (Steinegger and Söding, 2017) for clustering. The annotation generated functional descriptions, Gene Ontology (GO) terms, KEGG pathways, and COG categories for each predicted protein.

Secondary metabolite biosynthetic gene clusters (BGCs) were identified using the fungal version of antiSMASH (v8.0.4) through the online server (Blin et al., 2023). The analysis was performed using the genome assembly and gene annotation files with relaxed detection strictness. High Similarity Confidence domain architectures were manually inspected and verified using BLASTp against the UniProtKB database (Reviewed status). Comparative cluster alignments were performed with CAGECAT^2^ (van den Belt et al., 2023), while reference clusters were obtained from the MIBiG database https://mibig.secondarymetabolites.org/ (Zdouc et al., 2025).

Predicted protein sequences were also searched against the Pathogen-Host Interactions database (PHI-base) (version 4.19; last revised 09 February 2026) available at http://www.phi-base.org/index.jsp (Urban et al., 2025) to identify genes potentially involved in pathogen-host interactions. The PHI-base protein sequences were downloaded in FASTA format and formatted as a local BLAST protein database using BLAST+. The predicted proteins were then compared against this database using BLASTp with an E-value cutoff of 1 e^−5^. For further analysis, BLAST hits were filtered: only hits with a minimum amino acid identity of 40%, an alignment length of at least 100 amino acids, and an E-value ≤1e^−10^ were considered significant. These filtered results were used to identify putative homologs of experimentally characterized pathogenicity-related genes recorded in PHI-base.

### Comparative phylogenomics

Comparative phylogenomic analysis was performed to identify conserved genomic regions across fungal genomes using the PHAST toolkit (v1.9.7). A neutral model was inferred using phyloFit under a reversible nucleotide substitution framework and applied in phastCons and phyloP to detect conserved elements and evolutionary constraint (Hubisz et al., 2011).

Orthologous gene families were identified using OrthoFinder (v3.0.1b1) (Emms and Kelly, 2019) by comparing the predicted proteome of the studied strain with different species: *Fusarium oxysporum* Fo47 (NCBI accession number: GCF_013085055.1_ASM1308505v1), *Fusarium oxysporum* ME23 (NCBI accession number: GCA_030719095.1_ASM3071909v1), *Fusarium oxysporum* Fo5176 (NCBI accession number: GCA_025331925.1_ASM2533192v1), *Fusarium oxysporum* Fol4287 (NCBI accession number: GCF_000149955.1_ASM14995v2), *Fusarium oxysporum* II5 (NCBI accession number: GCA_031834405.1_ASM3183440v1), *Fusarium graminearum* (NCBI accession number: GCF_000240135.3_ASM24013v3), *Fusarium verticillioides* (NCBI accession number: GCF_000149555.1_ASM14955v1), and *Fusarium fujikuroi* (NCBI accession number: GCF_900079805.1_*Fusarium_fujikuroi*_IMI58289_V2).

To avoid redundancy, only the longest transcript per gene was retained for each proteome. Orthology inference was performed using the OrthoFinder workflow with multiple sequence alignment-based orthogroup inference.

### Production and extraction of fungal secondary metabolites

#### Liquid–liquid production and extraction

Fungal secondary metabolite production and extraction were based on the protocol described by Lebeau et al. (2019) with slight modifications (Supplementary Figure S1).

Fungal pre-cultures were grown in PDB medium at 30 °C, 160 rpm for 5 days. For inoculation, 1 mL of homogenized pre-culture was added to fresh PDB and incubated for 7 days under the same conditions. After fermentation, fungal biomass was separated by gauze filtration. Extracellular (EC) metabolites were extracted from the broth using ethyl acetate, with the solvent evaporated under vacuum. Mycelium and culture filtrate were lyophilized.

Dried biomass was weighed, ground, and intracellular (IC) metabolites were extracted by 24 h maceration with methanol and ethyl acetate. The solvent was then evaporated under vacuum. EC metabolites from the culture filtrate were also extracted by maceration with absolute methanol, and the solvent evaporated. All crude extracts were stored at 4 °C.

#### Solid–liquid production and extraction

The fungal strain 3RR was grown on 30 PDA Petri dishes (9 cm diameter) at 30 °C for 10 days. The contents of two dishes per endophyte were macerated for 24 h with either ethyl acetate or 100% methanol. The solvent was recovered via gauze and vacuum filtration, then evaporated under vacuum at 40 °C using a rotary evaporator. HPLC grade solvents were used.

### Biological activity assays

For biological activity studies, several laboratory biofilm-forming bacterial strains were used: Methicillin-resistant *Staphylococcus aureus* (MRSA) ATCC 33591 reference strain and *Staphylococcus epidermidis* S61 (Gram-positive) and *Escherichia coli* K12, *Pseudomonas aeruginosa* 2629 and *Serratia marcescens* C6LB (Gram-negative).

#### Antibacterial activity

Reference gram-positive and gram-negative bacteria were prepared by growing them in Mueller-Hinton medium (16–24 h, 37 °C) and adjusting their optical densities to 0.5 McFarland. Fungal extracts were dissolved in DMSO at a final concentration of 5%. Antibacterial activity was assessed using the agar disk diffusion method: wells were punched into Mueller-Hinton agar (MHA, composition in Supplementary Table S1), uniformly swabbed with bacterial inoculum, and then filled with fungal/bacterial extracts, chloramphenicol (positive control, 2.5 mg/mL), or DMSO (negative control). Disks of 9 mm diameter were used throughout the assay. Plates were incubated at 37 °C for 24 h, and inhibition zone diameters were measured to assess the resistance of the tested strains.

##### Determination of minimum inhibitory concentration

The Minimum Inhibitory Concentration (MIC) was determined in 96-well plates using a modified method. Mueller-Hinton Broth (MHB, composition in Supplementary Table S1) was added to most wells, with MHB containing 5% DMSO added to columns 1 and 2, and antibiotic to column 3. Sterilized test extracts were added to columns 4 and 5, followed by serial 1/2 dilutions from column 5 to 12. After adding MHB to column 2 onwards and inoculum, plates were incubated at 37 °C for 18–24 h. Growth was detected by adding 3-(4,5-dimethylthiazol-2-yl)-2,5-diphenyltetrazolium bromide (MTT), which turns blue-violet in the presence of viable bacteria (Supplementary Figure S2). The MIC was identified as the lowest concentration inhibiting growth. Assays were performed in duplicate.

##### Determination of minimum bactericidal concentration

The Minimum Bactericidal Concentration (MBC) was determined from MIC results by streaking wells without growth onto MH agar plates, incubated at 37 °C for 24 h. MBC was the first streak without growth (corresponding to the lowest concentration).

#### Antibiofilm activity

##### Anti-adherent and eradication activity

Fungal and bacterial extracts were evaluated for antibiofilm activity using modified methods by Abdelli et al. (2019) in 96-well plates. The antibiofilm activity was evaluated using anti-adherence and eradication assays. Two strains were selected based on their strong biofilm-forming ability: *S. aureus* (MRSA) ATCC 33591 (Zhu et al., 2026), previously isolated from a clinical environment and *S. epidermidis* S61, a strong biofilm producer identified in our prior work (Jardak et al., 2017).

For anti-adherence, extracts were serially diluted in Tryptic Soy Broth (TSB, composition in Supplementary Table S1) with DMSO, and plates were inoculated with bacteria and glucose (at a final concentration of 2.25%), then incubated for 18–24 h at 37 °C.

For eradication, biofilms were first formed for 48 h, then treated with extracts and re-incubated for 24 h at 37 °C. In both assays, after incubation, wells were washed, dried, and stained with crystal violet. Assays were performed in quadruplicate. Biofilms were disrupted with 33% acetic acid (v/v in distilled water to minimize polystyrene plate damage), and biofilm inhibition percentages were calculated using the following formulas:

$$\begin{array}{l} \text{Anti}-\text{adherent Activity}\;(\%)\\ =[(\mathrm{OD}\;\text{Control}-\mathrm{OD}\;\text{Treatment})/\mathrm{OD}\;\text{Control}]\times 100 \end{array}$$

$$\begin{array}{l} \text{Eradication Activity}\;(\%)\\ =[(\mathrm{OD}\;\text{Control}-\mathrm{OD}\;\text{Treatment})/\mathrm{OD}\;\text{Control}]\times 100 \end{array}$$

where OD Control is the optical density (570 nm) of untreated biofilms and OD Treatment is the optical density of biofilms treated with fungal extracts. The percentage inhibition indicates the reduction in biofilm mass compared to untreated controls.

##### Biofilm imaging

Biofilm visualization was performed using both optical and fluorescence microscopy. For optical microscopy, biofilms were grown on glass coverslips for 24 h, stained with crystal violet, and observed at 100X magnification using an OLYMPUS BX50 microscope. For fluorescence microscopy, 48-h biofilms in 24-well plates were stained with 0.1% acridine orange and observed at 40X magnification using a ZEISS epifluorescence microscope (excitation 460–490 nm, emission 520 nm). Images were analyzed with Zeiss Zen v.3.8 software.

### Analysis of *Fusarium* sp. (3RR) EC/EtOAc extract composition by gas chromatography–mass spectrometry

The ethyl acetate extracellular extract of *Fusarium* sp. (3RR) was analyzed by gas chromatography–mass spectrometry (GC-MS) using an Agilent 8,890 GC system equipped with a 5975C MSD. Samples were completely dried under nitrogen, derivatized with 30 μL pyridine and 60 μL *N, O*-bis(trimethylsilyl)trifluoroacetamide:trimethylchlorosilane [BSTFA: TMCS (99:1)], and incubated at 60 °C for 60 min. The derivatized sample was diluted, and 1 μL was injected. The column was an Agilent DB-5MS or HP-5MS UI (30 m × 0.25 mm i.d., 0.25 μm film thickness) with helium carrier gas at constant flow (1–1.5 mL/min). The oven temperature program started at 100 °C (hold 1 min), ramped to 190 °C at 20 °C/min (hold 15 min; if applicable), then to 280–325 °C at 4–5 °C/min (hold 3–5 min). The inlet was operated in splitless or split mode (10:1 ratio) at 250 °C (1.0 mL injection volume). The MSD was run in electron ionization (EI) scan mode (70 eV; m/z 30–600, threshold 150 counts; solvent delay 3 min) with source temperature at 230 °C and transfer line at 280 °C. Bioactive compounds were identified by comparing mass spectra to library databases.

### Statistical analysis

Descriptive statistical parameters including mean, standard deviation (SD), coefficient of variation (CV), standard error (SE), and 95% confidence intervals (95% CI) were determined for all assays. Data normality was evaluated using the Shapiro–Wilk test prior to inferential analyses. Comparisons between treated groups and untreated positive controls were performed using Student’s *t*-test, and reproducibility between replicates was assessed by intraclass correlation coefficient (ICC) analysis. Differences were considered statistically significant at p<0.05 .

All statistical analyses and graphical representations were performed using R software (version 4.5.2).

## Results

### Isolation of 3RR strain

After 7–15 days of incubation, endophytic microorganisms began to grow from the sterilized leaf fragments. A total of 60 endophytic microorganisms were isolated on solid medium after incubation from the Eppendorf tubes containing the culture medium and leaf fragments. Among them, a pigmented fungal strain named 3RR was identified as the primary subject of this study, upon which all subsequent analyses were conducted. Microscopic observations at 40 × and 100 × magnifications reveal long, thin, septate hyphae with smooth walls and limited branching. The colony exhibits a fluffy, cotton-like white appearance on the surface and a reddish to dark brown pigmentation on the reverse, consistent with typical Fusarium morphology (Supplementary Table S2).

### Phylogenetic analysis

The multiple sequence alignment analysis revealed similarities exceeding 98% with the ITS sequences of the closest fungal species. Phylogenetic analysis based on ITS sequences, demonstrated that fungal strain 3RR clusters robustly within the *Fusarium oxysporum* species complex, showing close evolutionary relatedness to reference *F. oxysporum* strains (Figure 1; bootstrap values indicated at nodes, 1000 replicates).

**Figure 1:**
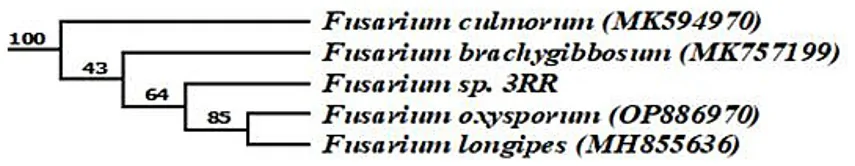
ITS-based phylogenetic analysis of *Fusarium* species. ITS (Internal Transcribed Spacer)-based phylogenetic reconstruction of field-isolated *Fusarium* strains and selected reference sequences retrieved from public databases. The tree was constructed using the neighbor-joining (NJ) method. Node robustness was assessed using 1,000 bootstrap replicates, and bootstrap values are indicated at branch nodes. The analysis confirms the taxonomic placement of isolate 3RR within the *Fusarium oxysporum* species complex and highlights its phylogenetic relationship with closely related strains.

### Sequencing data quality assessment

After quality control, trimming, and merging, the dataset was substantially reduced. The total number of sequences decreased to reach 2,463,817, retaining only ~23% of the original reads. Total bases dropped to 528 Mbp, corresponding to ~35% retention. Sequence lengths increased from 35–149 bp to 73–267 bp due to merging of paired-end reads and removal of short fragments. GC content remained stable at 47%, and no reads were flagged as poor quality, indicating high-quality input data (Supplementary Table S3).

For Nanopore sequencing, after preprocessing (filtering and quality trimming), 1,183,342 reads were retained (2.93 Gb), with a mean read length of 2,478 bp and a mean quality score of 14.3. The N50 increased 2,880 bp, indicating enrichment of longer, higher-quality reads. The fraction of reads above Q10 increased from 86.5% in raw reads to 99.8% in processed reads, while reads above Q12 rose from 67.1 to 93.0%, reflecting successful removal of low-quality reads (Supplementary Figure S3).

K-mer analysis across a range of k-mer sizes (21–101) revealed consistent genome size estimates, indicating stable and reliable measurements. Genome size estimates gradually increased from 43.18 Mb at k = 21 to 45.91 Mb at k = 101 (Supplementary Figure S4), with the most stable values observed between k = 21 and k = 71 (43.18–44.11 Mb). This convergence suggests that the sequencing data is of sufficient quality and that k-mer sizes in the 21–31 range are optimal for downstream assembly. Minor increases at larger k-mer sizes likely reflect the influence of repetitive regions in the genome. Overall, the analysis indicates a genome of approximately 44 Mb, with low heterozygosity and moderate repetitive content, providing a robust baseline for assembly parameter optimization.

### Hybrid assembly using SPAdes

A total of 14.1 million reads were assembled using SPAdes, resulting in 1043 contigs. The largest contig was 2.37 Mb and the total assembly length was 42.86 Mb. Assembly continuity was indicated by an N50 of 0.74 Mb and N90 of 0.041 Mb (L50 = 16, L90 = 99). The genome had a GC content of 47.86% and an average coverage depth of 46×, with 99.84% of bases covered at ≥10×. Per-base quality was high, with only 5.08 Ns per 100 kbp.

Taxonomic classification of the assembled contigs using Kraken2 (v2.1.6) confirmed that the majority of sequences belong to fungal taxa within the genus *Fusarium oxysporum*, with no substantial evidence of contamination from non-fungal organisms.

### Secondary scaffolding

The hybrid genome assembly was further improved through secondary scaffolding using multiple reference genomes, which substantially enhanced contiguity and structural coherence. The final scaffolded assembly comprised 12 contigs with a total genome size of approximately ~41.1 Mb, including a largest contig of 7.58 Mb. Assembly quality metrics showed a marked improvement compared to the primary hybrid assembly, with the N50 increasing from 738 kb to 3.52 Mb and the L50 decreasing from 16 to 4, indicating a significantly more contiguous and better-resolved genome structure (Figure 2). These improvements provide stronger support for downstream functional annotation and genome interpretation.

**Figure 2:**
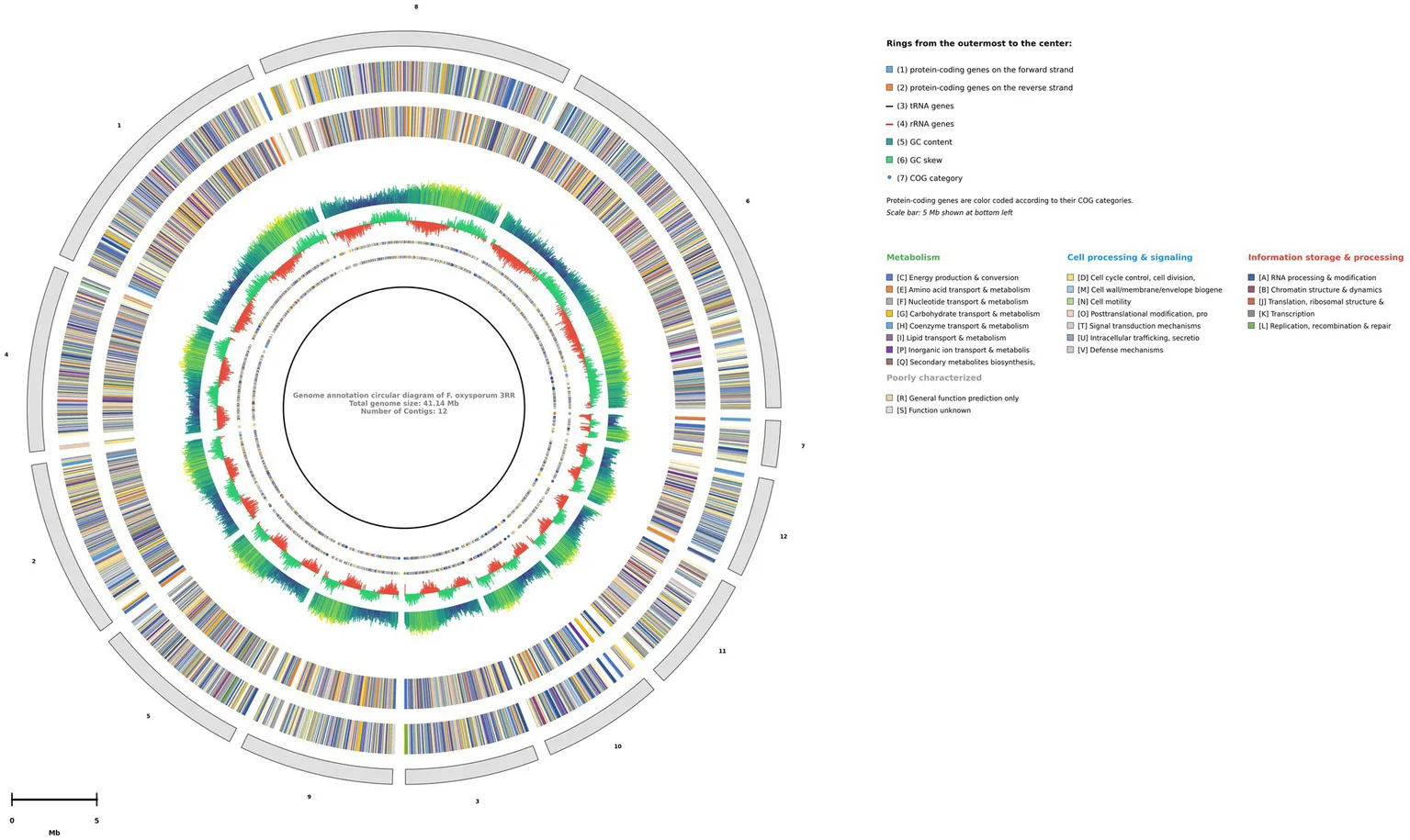
Circular genome annotation of *Fusarium oxysporum* 3RR. Circular genome map of *Fusarium oxysporum* strain 3RR illustrating genome-wide functional annotation. Protein-coding genes are annotated and classified according to KOG (euKaryotic Orthologous Groups) functional categories, including metabolism, cellular processes and signaling, and information storage and processing. The outer rings represent gene density and functional distribution across genomic regions, while inner tracks display GC content and GC skew. The scale bar corresponds to 5 Mb, providing genomic positional reference across the assembled contigs.

Overall, secondary scaffolding reduced fragmentation and produced chromosome-scale scaffolds, facilitating downstream analyses and comparative genomics. Coverage and completeness remained high (>98% bases covered ≥1×), consistent with the high-quality hybrid assembly.

BUSCO analysis of the Ragout-scaffolded assembly against the fungi_odb12 dataset showed 95% complete BUSCOs (94.5% single-copy, 0.5% duplicated), with 0.2% fragmented and 4.8% missing, indicating a highly complete fungal genome. Of the 1,066 complete BUSCOs, 196 (18.4%) contained internal stop codons, reflecting potential gene prediction artifacts from Miniprot (Table 1).

**Table 1:** SPAdes hybrid assembly statistics and BUSCO assessment.

Metrics		Value
QUAST statistics	Number of contigs	12
	Total length (Mb)	41.14
	Largest contig (Mb)	7.58
	N50 (Mb)	3.52
	N90 (Mb)	2.20
	L50	4
	L90	10
	GC (%)	48.18
BUSCO statistics	Complete (C)	95.0%
	Single-copy (S)	94.5%
	Duplicated (D)	0.5%
	Fragmented (F)	0.2%
	Missing (M)	4.8%
	Total BUSCOs	1,122

Mb: Mega base, N50: Length of the contig at which 50% of the genome assembly is contained in contigs of that length or longer, N90: Length of the contig at which 90% of the genome assembly is contained in contigs of that length or longer, L50: Minimum number of contigs whose combined length makes up 50% of the total assembly, L90: Minimum number of contigs whose combined length makes up 90% of the total assembly, GC (%): Percentage of guanine and cytosine nucleotides in the genome sequence.

### Functional analysis

#### Functional annotation

A total of 2,103,384 bp (5.11%) of the genome was masked as repetitive DNA, including 4.44% interspersed repeats, mainly LTR retroelements (1.03%) dominated by Gypsy/DIRS1 elements (0.89%), and DNA transposons (0.71%), particularly Tc1-IS630-Pogo (0.48%). A notable proportion of repeats (2.35%) remained unclassified, while additional elements such as simple repeats, satellites, small RNA, and low-complexity regions were also detected (Supplementary Figure S5).

Gene prediction was performed on the *Fusarium oxysporum* genome using BRAKER3 with protein evidence from a curated Fungi database. A total of 11,738 genes were predicted; each assigned a unique gene identifier. Examination of the annotation in the GTF file revealed 37,626 exons, corresponding to an average of approximately 3.2 exons per gene. The predicted coding sequences amounted to 12,547 CDS entries, which matched the number of predicted proteins, indicating that each coding sequence translated to a single protein product.

Functional annotation using InterProScan identified matches for 11,664 protein sequences (93% of the predicted proteome), with 577,904 individual domain or signature hits across multiple databases. The distribution of annotations by module is represented in Supplementary Table S4.

A total of 4,416 unique Pfam domains were identified in the analyzed proteome. The most abundant domains indicate a predominance of transcription factors, transporters, and catalytic proteins. The distribution of the top Pfam domains is presented in Supplementary Figure S6.

Functional annotation of the predicted proteome was performed using multiple databases through the eggNOG-mapper pipeline. A total of 12,132 proteins were assigned to eggNOG orthologous groups. The COG functional classification showed that 8,995 unigenes were distributed across 10 major COG categories. The analysis revealed that a large portion of genes fall into the category of “function unknown,” followed by post-translational modifications, carbohydrate metabolism, secondary metabolism, amino acid metabolism, and transcription. Other significant categories included Intracellular trafficking, Energy production, Signal transduction, and translation (Figure 3). These results suggest that genes in these categories are likely critical for fundamental cellular processes and overall organismal development.

**Figure 3:**
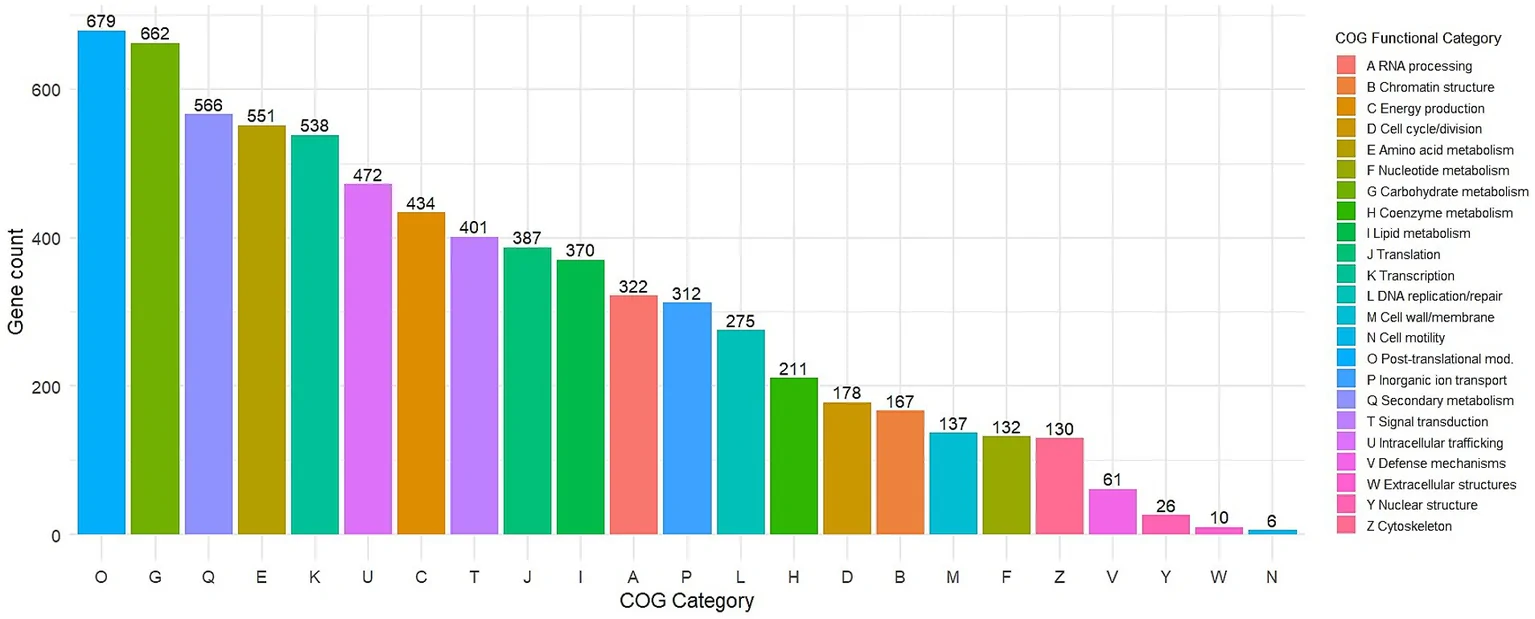
COG functional classification of predicted genes. Distribution of predicted protein-coding genes assigned to COG (Clusters of Orthologous Groups) functional categories. Categories include cellular processes and signaling, metabolism, and information storage and processing, with further sub-functional divisions (e.g., energy production, transcription, replication, and repair). The x-axis represents COG functional classes (O, G, Q, E, K, U, C, T, J, I, A, P, L, H, D, B, M, F, Z, V, Y, W, N), while the y-axis indicates gene counts per category, reflecting the functional complexity of the genome.

Across all genes, a total of 339,884 GO entries were recorded, encompassing 8,902 unique GO terms. Breaking down by GO category, cellular process and metabolic process emerged as the most represented activities in Biological Process (BP). The most frequent terms in the Cellular Component (CC) category are intracellular anatomical structure and intracellular organelle. As for Molecular Function (MF), catalytic activity and binding are the leading functional categories. This pattern highlights the prevalence of enzymes and binding proteins, consistent with a transcriptome geared toward diverse biochemical and regulatory activities.

The distribution and frequency of the top GO terms across each ontology class are illustrated in Figure 4.

**Figure 4:**
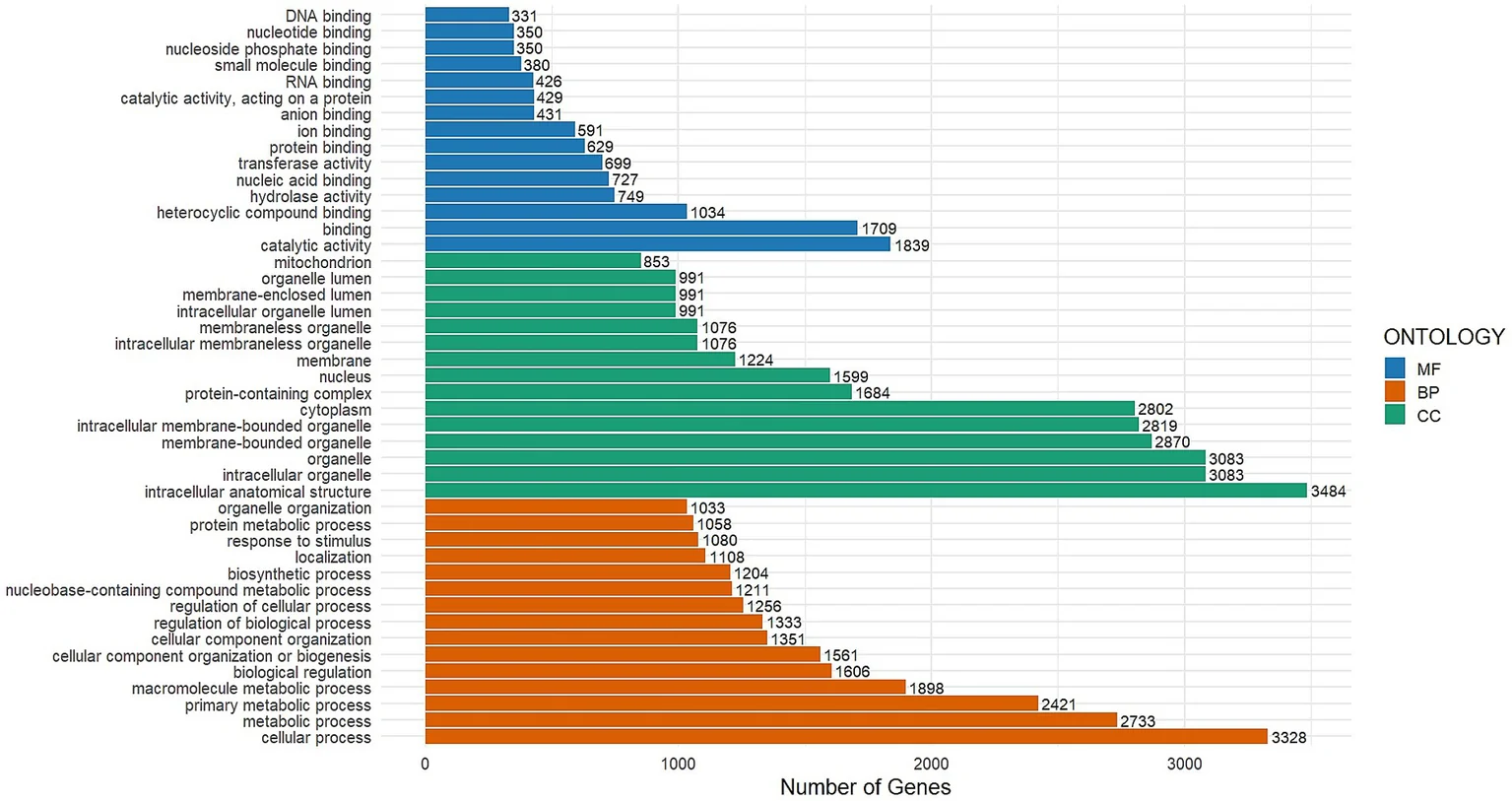
Gene ontology (GO) functional classification. GO-based functional annotation of predicted genes categorized into three main domains: Molecular Function (MF), Biological Process (BP), and Cellular Component (CC). Each bar represents the number of genes assigned to specific GO subcategories, providing insights into enzyme activity, biological pathways, and subcellular localization. The distribution highlights genome-wide functional diversity and supports downstream interpretation of metabolic and regulatory capabilities.

The functional annotation revealed extensive mapping to KEGG terms. A total of 3,317 unique KEGG Orthology (KO) terms were identified, with the most abundant including yeast amino acid transporter. KEGG pathway mapping revealed that a substantial number of unigenes participate in fundamental cellular and metabolic processes, with the highest enrichment observed in general metabolic pathways (map01100) and the biosynthesis of secondary metabolites (map01110) (Figure 5).

**Figure 5:**
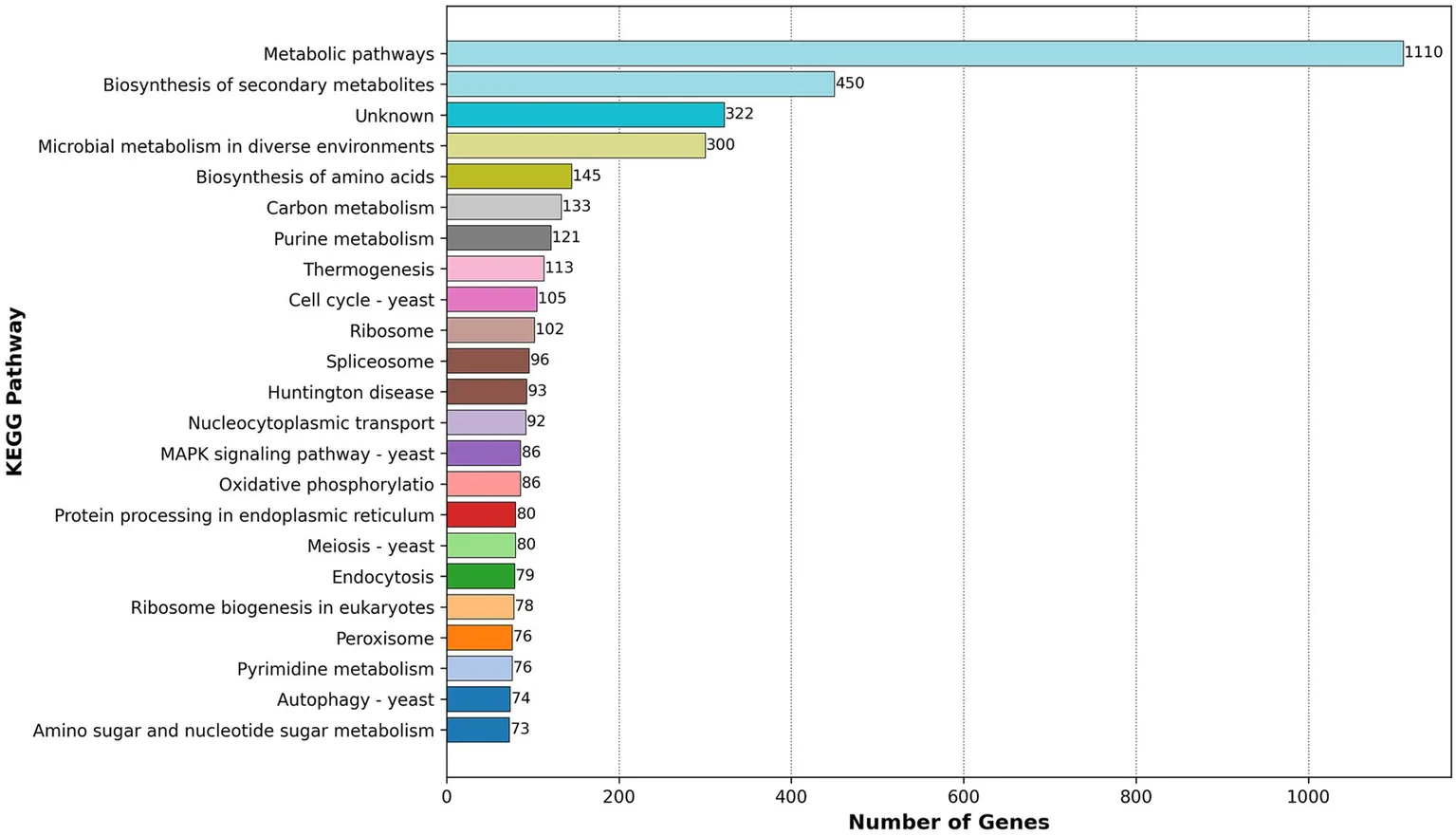
KEGG pathway functional distribution. Functional classification of predicted genes mapped to KEGG (Kyoto Encyclopedia of Genes and Genomes) pathways. The analysis highlights genes involved in key biological systems, including primary metabolism, secondary metabolite biosynthesis, environmental information processing, and cellular processes. The distribution reflects the metabolic versatility of *F. oxysporum* 3RR and its potential involvement in biosynthesis of bioactive compounds.

These results demonstrate that the annotated genes are involved in a broad spectrum of metabolic and regulatory functions, providing a comprehensive overview of the organism’s functional potential.

A comprehensive analysis of enzyme classification revealed 1,063 unique EC numbers distributed across all six major enzyme classes Hydrolases (35.7%) and Transferases (33.6%) dominated the dataset, collectively accounting for around 69% of all enzymes. Notable variation was observed in the frequency distribution of specific EC numbers (Supplementary Figure S7) with EC 2.7.11.1 (protein kinases) being the most prevalent, followed by EC 3.6.4.13 (DNA helicases) and EC 3.6.4.12 (RNA/DNA helicases). The high prevalence of kinases and helicases suggests significant involvement of phosphorylation and nucleic acid processing activities in the studied biological system.

Analysis of the carbohydrate-active enzyme (CAZy) families revealed that glycoside hydrolases (GH) were the most abundant, with over 100 genes identified in the genome. This was followed by glycosyltransferases (GT), which accounted for 61 genes. Other families were present at lower counts (Figure 6).

**Figure 6:**
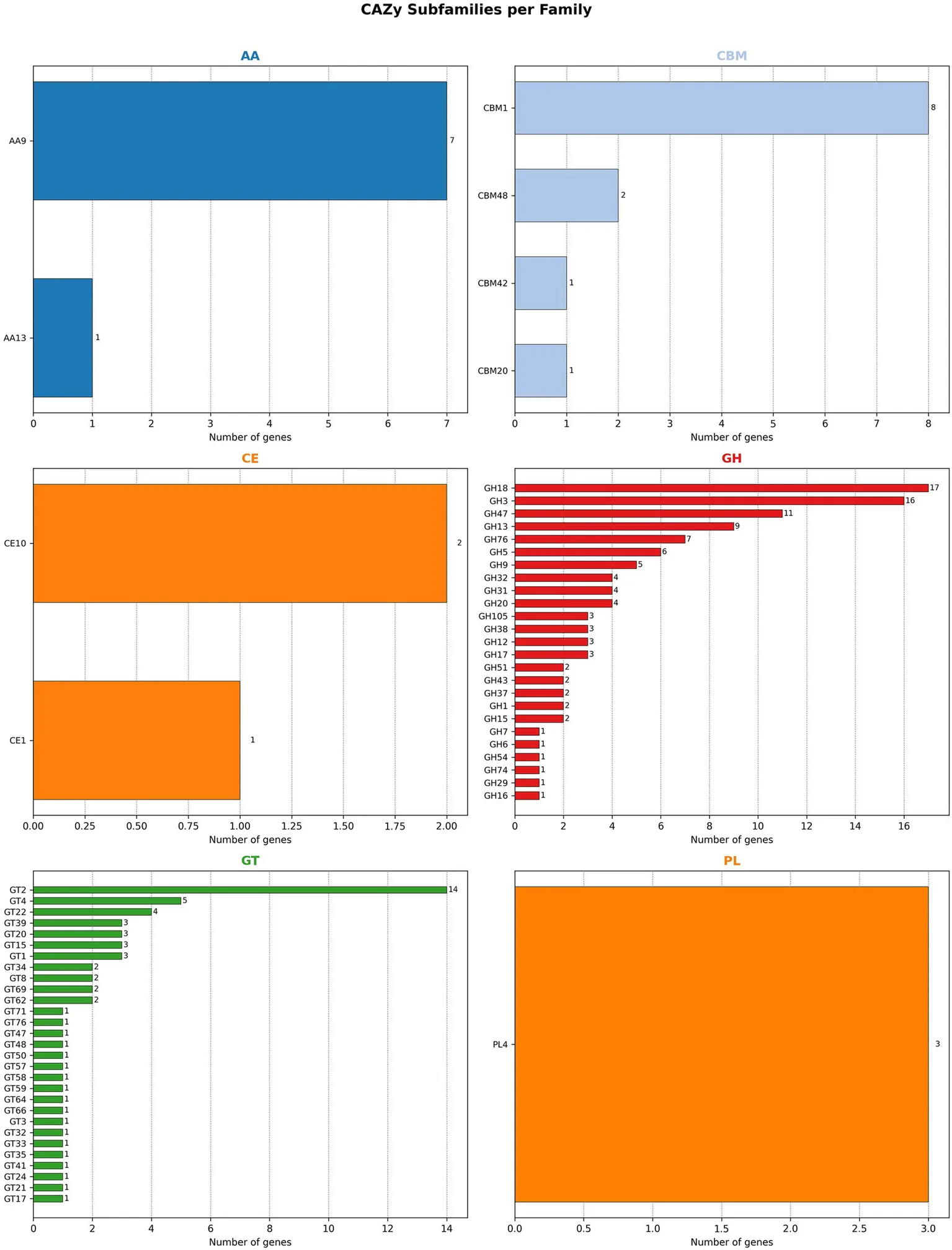
CAZyme family distribution analysis. Genome-wide identification and classification of carbohydrate-active enzyme (CAZyme) genes in *F. oxysporum* 3RR. CAZymes are grouped into six major functional families: Glycoside Hydrolases (GH), Glycosyltransferases (GT), Carbohydrate Esterases (CE), Polysaccharide Lyases (PL), Auxiliary Activities (AA), and Carbohydrate-Binding Modules (CBM). This distribution reflects the organism’s capacity for carbohydrate degradation, host interaction, and biomass utilization.

These results indicate a strong potential for carbohydrate metabolism and modification.

The analysis of CAZysubfamilies shows that within the GT family, the GT2 subfamily (includes cellulose synthases, chitin synthases, and related transferases) is the most abundant, indicating a strong expansion of this glycosyltransferase type in the genome.

within the GH family, several subfamilies are prominent: GH18 (chitinases and related enzymes) and GH3 (exoglycosidases and related enzymes) are the most frequent, suggesting that enzymes in these subfamilies may play key roles in carbohydrate degradation and remodeling processes in this organism.

Overall, the distribution of subfamilies highlights that certain subfamilies dominate their respective families, reflecting potential specialization or expansion of these enzymatic functions.

GH: Glycoside Hydrolases, Glycosyl Transferases, CBM: Carbohydrate-Binding Modules, AA: Auxiliary Activities, CE: Carbohydrate Esterases, PL: Polysaccharide Lyases.

#### Investigation of secondary metabolite biosynthetic gene clusters

Secondary metabolite biosynthetic gene clusters (BGCs) were predicted using antiSMASH which identified 36 putative clusters distributed across the 12 contigs of the *Fusarium oxysporum* genome. The detected clusters belong to several major biosynthetic classes, including non-ribosomal peptide synthetases (NRPS and NRPS-like), terpenes, polyketide synthases (PKS), betalactones, and isocyanides (Figure 7).

**Figure 7:**
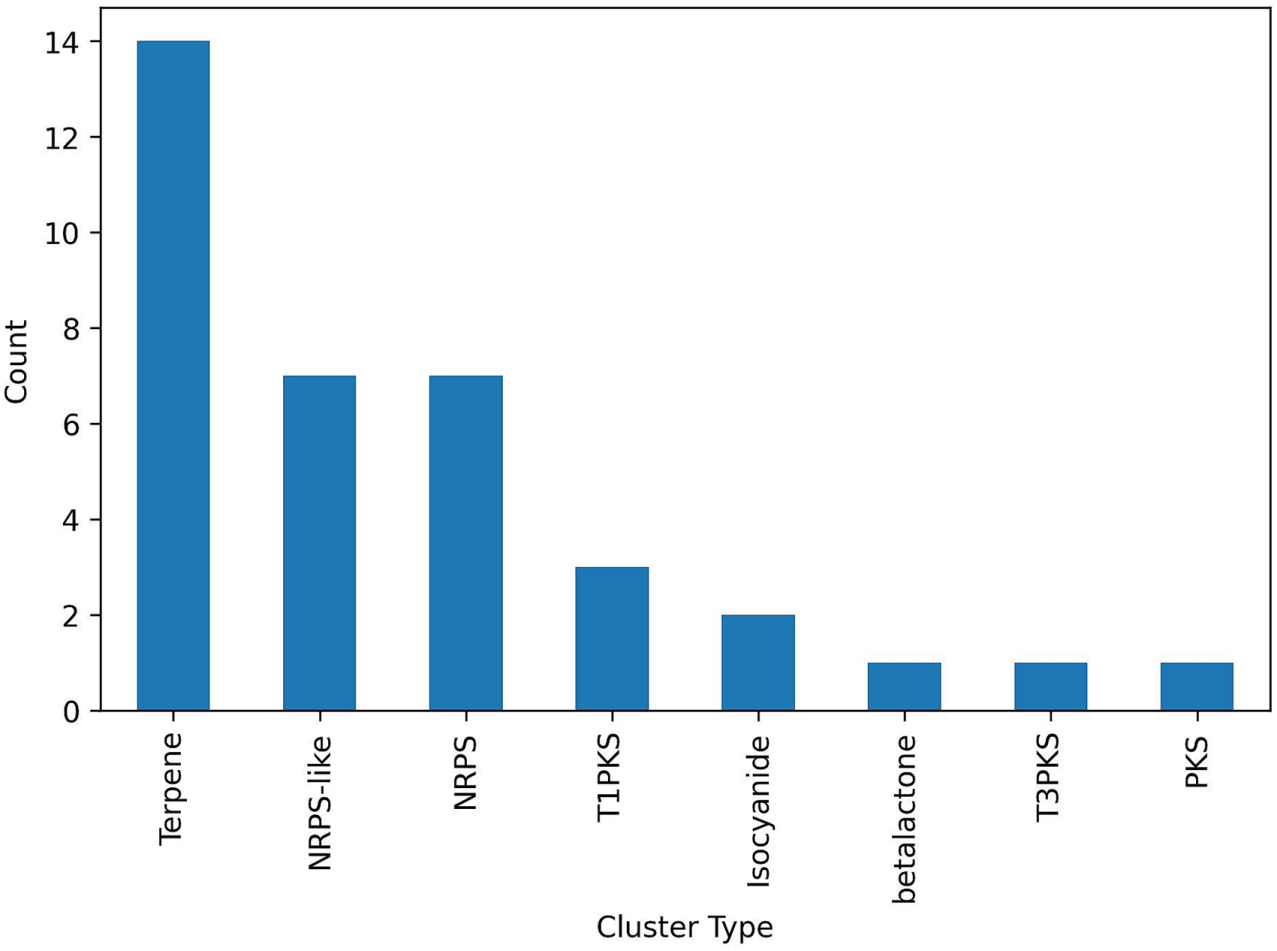
Secondary metabolite biosynthetic gene cluster (BGC) analysis. Prediction and classification of secondary metabolite biosynthetic gene clusters (BGCs) identified using genome mining approaches. Eight major cluster types were detected, including terpene, non-ribosomal peptide synthetase (NRPS), NRPS-like, Type I/II/III polyketide synthases (T1PKS, T2PKS, T3PKS), isocyanide, betalactone, and hybrid PKS systems. These clusters suggest a strong biosynthetic potential for antimicrobial and bioactive compound production.

NRPS-related clusters and terpene clusters were the most abundant categories. Notably, six clusters were classified as high confidence predictions (9.5% of total clusters), representing robust and well-characterized biosynthetic pathways. These HIGH confidence clusters comprise three polyketide/NRPS-derived classes (regions 1.3, 3.1, and 9.1) and three sesquiterpene clusters (regions 2.1, 10.3, and 12.4) (Supplementary Table S5).

The largest and most architecturally complex high confidence cluster (Region 9.1, 114.6 kb) encodes a hybrid biosynthetic pathway containing Type I PKS, NRPS-like, and isocyanide domains, predicted to synthesize aurofusarin, a well-characterized mycotoxin with known roles in Fusarium virulence and host pathogenicity. Additional pathogenicity-relevant clusters include a Type I NRPS cluster (Region 1.3, 63.8 kb) predicted to synthesize choline-derived amino acid compounds, essential for membrane biogenesis and osmotic adaptation during fungal-plant interactions, and a Type I PKS cluster (Region 3.1, 67.1 kb) encoding oxyjavanacin biosynthesis. The three sesquiterpene clusters (Regions 2.1, 10.3, and 12.4, ranging from 31–32 kb) are predicted to produce clavaric acid, koraiole, and α-acorenol—volatile secondary metabolites implicated in host recognition, toxin production, stress response, and root colonization signaling.

Several additional clusters showed low similarity to biosynthetic pathways in the MIBiG database for previously characterized compounds including squalestatin S1, mangicol A, ilicicolin H, orcinol/orsellinic acid, and T-2 toxin, suggesting the potential presence of novel or divergent biosynthetic pathways.

#### Pathogen host interactions

A total of 5,540 predicted proteins showed similarity to PHI-base entries based on BLASTp searches. After applying stringent filtering criteria, 2,673 proteins were retained as high-confidence homologs of experimentally characterized pathogen–host interaction genes, matching 3,959 unique PHI-base proteins. The overall sequence similarity was relatively high, with an average identity of 62.3%, and the alignments spanned an average length of 439 amino acids, indicating substantial coverage of the homologous regions.

PHI-base annotation revealed distinct functional categories among matched genes. Genes associated with reduced virulence when disrupted constitute the largest category, representing 50.6% of total hits, distributed across 1,565 unique genes in our dataset. This was followed by genes with unaffected pathogenicity (30.9%), and loss-of-pathogenicity phenotypes (10.2%) (Figure 8).

**Figure 8:**
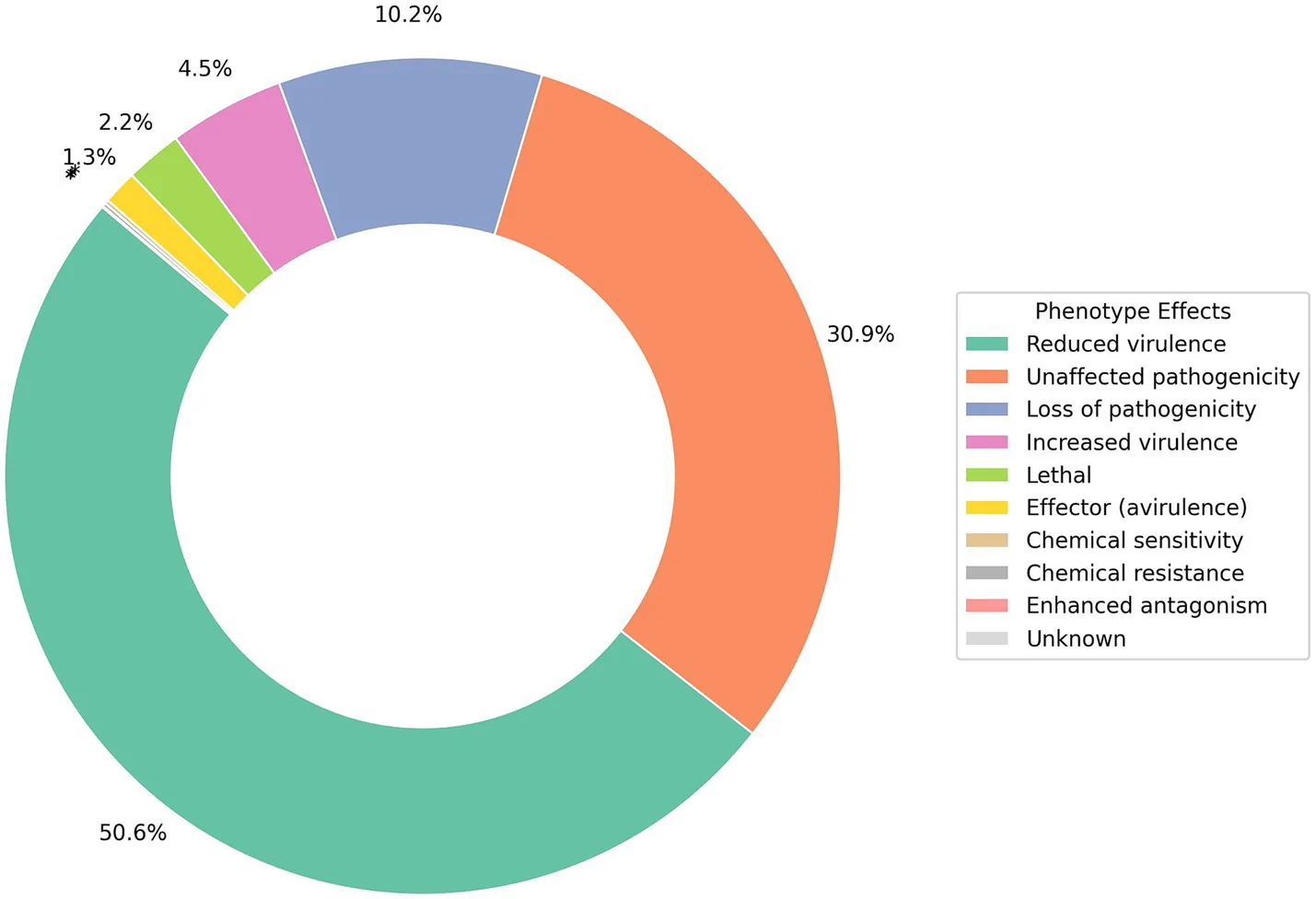
PHI-base phenotype effect distribution. Distribution of predicted gene functions based on PHI-base (Pathogen-Host Interaction database) annotation. Genes are categorized according to their predicted effects on pathogen virulence, including reduced virulence, unaffected pathogenicity, lethal phenotype, and increased virulence. The proportional representation highlights the role of annotated genes in host-pathogen interactions and potential virulence mechanisms.

Intriguingly, 186 genes exhibited dual phenotypes, being associated with both reduced and increased virulence depending on the pathogenic context or host species. This bidirectional phenotypic association suggests functional pleiotropy or context-dependent virulence contributions, warranting further investigation.

### Comparative phylogenomics

Evolutionarily conserved regions were identified using the PHAST package based on the multiple genome alignment. Using phastCons, a total of 3,914 conserved elements were detected, covering 1.003 Mb of the genome. The predicted conserved elements had a mean length of 256 bp, with sizes ranging from 40 bp to 3,649 bp and a median length of 166 bp.

To assess conservation at the nucleotide level, phyloP analysis was performed across 6,487,135 sites. Among these, 5,155,955 sites showed positive scores, indicating evolutionary conservation, whereas 1,309,380 sites exhibited negative scores, suggesting accelerated evolution relative to the neutral model. Together, these analyses reveal a substantial number of conserved genomic regions and nucleotide sites under evolutionary constraint across the analyzed genomes.

Orthologous relationships among the analyzed species were inferred using OrthoFinder, enabling the identification of shared and lineage-specific gene families across six Fusarium genomes.

A large proportion of genes from all analyzed species were successfully assigned to orthogroups, highlighting a high level of gene conservation across the genomes. In our strain, 12,077 out of 12,547 genes (96.3%) were clustered into orthogroups, while 470 genes (3.7%) remained unassigned. In total, 10,874 orthogroups contained at least one gene from our strain, indicating substantial participation in shared gene families among the studied species.

Species-specific orthogroups were also identified, representing gene families unique to individual genomes. Our strain possessed 106 species-specific orthogroups, comprising 245 genes, which correspond to approximately 2.0% of its predicted proteome.

Taken together, the findings suggest strong genomic conservation across the analyzed *Fusarium* species, alongside a limited set of unique genes that likely underpin species-specific traits and adaptive differences.

Gene duplication events were also inferred to investigate patterns of gene family expansion across the analyzed *Fusarium* genomes. Analysis of gene duplications across the species tree revealed that our strain has a total of 1,050 gene duplication events, all of which are supported at ≥50% confidence, indicating that these duplications are highly reliable. Compared to other *Fusarium* species in the analysis, such as Fo5176 (3,560 duplications) and II5 (6,832 duplications), our strain exhibits a moderate level of gene duplication. These results suggest that while duplication has contributed to its genome evolution, the extent of gene family expansion in our strain is less pronounced than in some closely related lineages.

Overall, these results highlight both ancestral and lineage-specific gene duplication events, contributing to the diversification of gene families across the analyzed *Fusarium* genomes.

Functional annotation of 106 species-specific orthologs using eggNOG and InterPro provided a comprehensive overview of their functional roles and structural features. Overall, InterPro achieved higher coverage, identifying conserved domains in 80.2% of proteins, while eggNOG assigned functional categories to 54.7% based on orthology. Nearly half of the dataset received support from both databases, highlighting the complementarity and reliability of the combined annotation approach.

COG-based classification revealed a diverse functional landscape dominated by metabolic and regulatory processes. Secondary metabolite biosynthesis emerged as a key category, representing 13.8% of annotated proteins, and included enzymes associated with specialized metabolism. Transcription-related proteins accounted for 8.6%, indicating the presence of species-specific regulatory mechanisms. A substantial proportion of proteins (31.0%) were assigned to the “function unknown” category, suggesting the presence of novel or highly diverged genes. Additional categories encompassed core metabolic processes, including energy production, amino acid metabolism, and cellular functions, reflecting broad biological roles.

InterPro analysis identified a total of 559 domain hits corresponding to 122 unique entries, providing a comprehensive overview of protein domain architecture across the dataset. These annotations were supported by multiple complementary databases. Notably, intrinsically disordered regions accounted for a substantial proportion of the annotations (29.2%), indicating that many proteins likely possess flexible conformations associated with regulatory and signaling functions (Supplementary Figure S8A). This high level of disorder, combined with the widespread presence of ankyrin repeats, further suggests that a significant fraction of these proteins may act as interaction hubs within cellular networks. The distribution of detected domains highlights a predominance of ankyrin repeats and Zn(2)Cys(6) fungal-type DNA-binding domains among the most abundant families, alongside key contributions from transcriptional regulators, transporters, and metabolic enzymes (Supplementary Figure S8B).

Overall, the frequent occurrence of multi-domain architectures, often combining regulatory, catalytic, and interaction modules, supports the presence of functionally complex proteins, reinforcing the conclusion that these orthologs are highly specialized and likely contribute to species-specific regulatory and metabolic adaptations.

Combined analysis demonstrated that most orthologs could be functionally characterized, with only a minority lacking domain or orthology-based annotations. The integration of eggNOG and InterPro data enabled robust functional inference, linking conserved domains with biological pathways and molecular functions.

### Production and extraction of secondary metabolites produced by 3RR

Two extraction solvents with different polarities were strategically selected to optimize secondary metabolite recovery. Ethyl acetate (EtOAc, semi-polar) preferentially extracts lipophilic compounds including fatty acids, phenolic compounds, and terpenoids, whereas methanol (MeOH, polar) extracts hydrophilic metabolites and carbohydrates. This dual-solvent approach ensures comprehensive coverage of the metabolite spectrum and avoids bias inherent to single-solvent extraction. The rationale is supported by the differential extraction yields shown in Supplementary Table S5 and the distinct bioactivity profiles reported in Supplementary Table S6, which demonstrate that each solvent recovers unique metabolite populations with different antimicrobial and anti-biofilm properties.

#### Yield of solid/liquid extraction of secondary metabolites

After 10 days of incubation of fungal endophytes on potato dextrose agar, metabolites were extracted using ethyl acetate or methanol. Yields were measured as extract quantity (in mg) per cm^2^ of PDA and results were presented in Supplementary Figure S9.

#### Yield of liquid/liquid extraction of metabolites

Liquid–liquid extraction of fungal secondary metabolites was performed on the culture of the endophytic strain 3RR. Metabolite production yields were calculated as the weight of the obtained extract (ethyl acetate or methanol) per volume of culture and per mycelium dry weight for both solvents. The yields are summarized in Supplementary Table S6.

### Biological characterization of the extracts obtained from 3RR

#### Evaluation of antibacterial activity using the agar diffusion method

The agar diffusion method is used to determine the diameter (in mm) of the inhibition zone of bacterial growth for each fungal extract. The results of the antimicrobial activity of the different secondary metabolites, Extra Cellular (EC) and Intra Cellular (IC), are presented in Supplementary Table S7.

A significant inhibition zone was observed against the four bacteria (except *P. aeruginosa*) with the EC/EtOAc extract from strain 3RR. The *S. epidermidis* strain showed the largest inhibition diameter (32–34 mm) compared to the others. The IC/EtOAc extract (3RR) also produced significant inhibition zones, albeit smaller (10–12 mm).

MIC and MBC values (Table 2) showed that all tested bacterial strains were susceptible to the extracts, with varying levels of sensitivity. The EC/EtOAc (3RR) extract exhibited the strongest antibacterial activity, particularly against *S. aureus*, *S. epidermidis*, and *E. coli*. Among the strains, *S. epidermidis* was the most sensitive, while *S. marcescens* showed comparatively lower susceptibility.

**Table 2:** Antibacterial activity of fungal extracts against 5 bacterial strains with the microtitration method.

Fungal extracts	Bacterial strains									
	*Staphylococcus aureus*		*Staphylococcus epidermidis*		*Pseudomonas aeruginosa*		*Escherichia coli*		*Serratia marcescens*	
	MIC*	MBC*	MIC*	MBC*	MIC*	MBC*	MIC*	MBC*	MIC*	MBC*
EC/EtOAc (3RR)	0.19	0.39	0.19	0.39	12.5	50	0.19	50	3.125	50
IC/EtOAc (3RR)	0.39	6.25	0.39	1.56	25	50	25	50	12.5	50
EC/MeOH (3RR)	25	25	0.39	12.5	25	50	25	50	12.5	50
IC/MeOH (3RR)	25	25	12.5	50	12.5	50	12.5	50	12.5	50

* Expressed in (mg/ml), EC/EtOAc (3RR), Extracellular fraction extracted with ethyl acetate from strain 3RR; IC/EtOAc (3RR), Intracellular fraction extracted with ethyl acetate from strain 3RR; EC/MeOH (3RR), Extracellular fraction extracted with methanol from strain 3RR; IC/MeOH (3RR), Intracellular fraction extracted with methanol from strain 3RR; MIC, Minimum Inhibitory Concentration.

#### Antibiofilm activity of fungal extracts

The antibiofilm activity (anti-adhesive and eradication) of the EC/EtOAc (3RR) and IC/EtOAc (3RR) extracts was evaluated based on their MIC results, using *S. epidermidis* (S61) and *S. aureus* (SARM) as biofilm models, having the strongest antibacterial effect. Antibiofilm activities were assessed against both forming biofilms (anti-adhesive activity) and mature biofilms (eradication activity).

##### Against *Staphylococcus epidermidis* (S61)

Adhesion assays were performed using concentrations ranging from the MIC (0.19 mg/mL) to 0.00075 mg/mL. The results showed that the EC/EtOAc (3RR) extract exhibited strong anti-adhesive activity against *S. epidermidis* S61 biofilm formation. At 0.19 mg/mL, the extract inhibited biofilm formation by 96.11% ± 2.78% (CV = 2.90%). A progressive decrease in anti-adhesive activity was observed at 0.095 mg/mL, 0.048 mg/mL, and 0.024 mg/mL, with inhibition rates of 84.35, 82.85, and 81.91%, respectively. Moderate inhibition was maintained at lower concentrations, reaching 69.91% at both 0.003 and 0.0015 mg/mL, while the lowest tested concentration (0.00075 mg/mL) still exhibited 55.89% inhibition. Statistical analysis confirmed a highly significant reduction in biofilm formation compared with the untreated positive control (*p* < 0.001).

In contrast, the IC/EtOAc (3RR) extract demonstrated lower anti-adhesive activity. At 0.19 mg/mL, biofilm inhibition reached 77.19% ± 8.03% (CV = 10.40%), followed by a progressive decline at decreasing concentrations. Inhibition rates decreased to 64.95% at 0.095 mg/mL, 59.92% at 0.048 mg/mL, and 57.44% at 0.024 mg/mL. At the lowest concentrations, activity markedly declined, reaching 26.78% at 0.003 mg/mL and 8.23% at 0.0015 mg/mL. Despite this reduction, the inhibition observed at 0.19 mg/mL remained statistically significant compared with the untreated control (*p* < 0.001) (Figure 9A).

**Figure 9:**
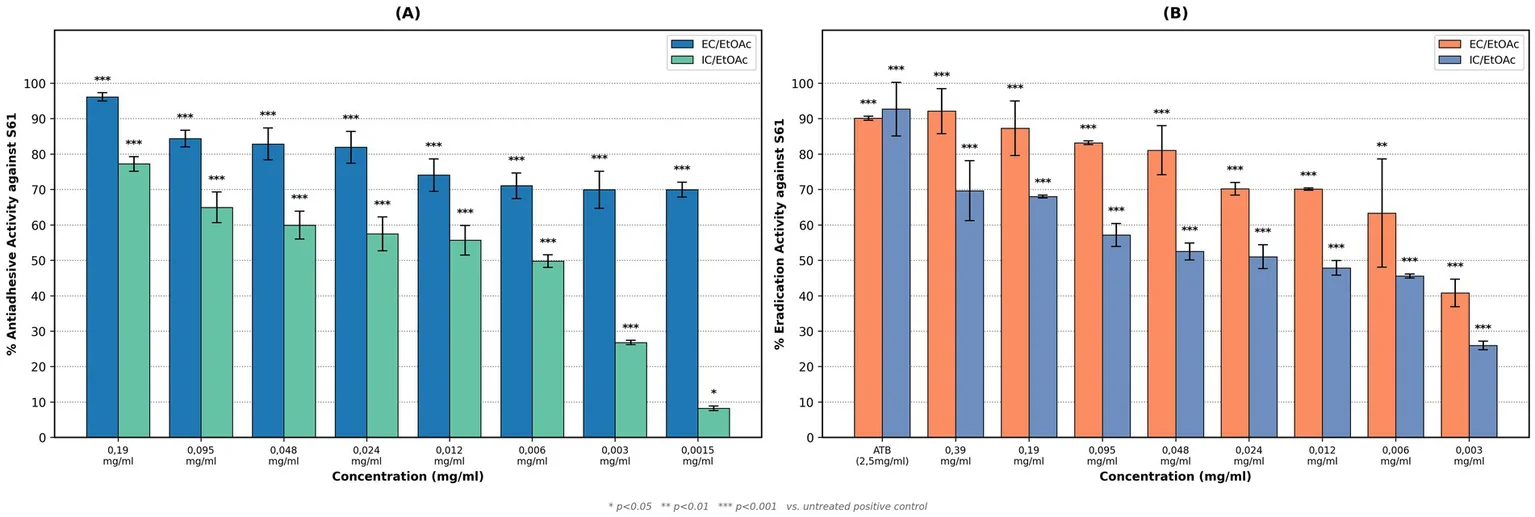
Antibiofilm activity against *S. epidermidis*. Comparative antibiofilm activity of extracellular (EC/EtOAc) and intracellular (IC/EtOAc) extracts from *Fusarium* sp. 3RR against *S. epidermidis*. Biofilm inhibition was assessed across a concentration gradient (0.19–0.0015 mg/mL) under standardized *in vitro* conditions. Anti-adhesive activity **(A)** represents inhibition of initial bacterial attachment, while eradication activity **(B)** reflects disruption of pre-formed mature biofilms. Antibiotic control (ATB, 2.5 mg/mL) was included as a positive reference. Data represent mean ± SD of triplicate independent experiments.

The efficacy of the EC/EtOAc (3RR) and IC/EtOAc (3RR) fungal extracts in eradicating mature *S. epidermidis* S61 biofilms was evaluated at concentrations ranging from 0.78 mg/mL to 0.0015 mg/mL. The EC/EtOAc (3RR) extract showed the highest eradication activity at 0.39 mg/mL, with a maximum inhibition rate of 92.09% ± 5.87% (CV = 6.37%). A concentration-dependent reduction in activity was subsequently observed, with inhibition rates of 87.29, 83.16, and 81.04% at 0.19, 0.095, and 0.048 mg/mL, respectively. Lower concentrations retained moderate activity, reaching 63.33% at 0.006 mg/mL and 40.79% at 0.003 mg/mL, while the lowest concentration tested (0.0015 mg/mL) showed 23.15% inhibition.

Comparatively, the IC/EtOAc (3RR) extract exhibited weaker biofilm eradication activity. Maximum inhibition was observed at 0.78 mg/mL, with an eradication rate of 83.22% ± 6.91% (CV = 8.31%). Activity progressively decreased with dilution, reaching 69.65% at 0.39 mg/mL, 68.01% at 0.19 mg/mL, and 57.17% at 0.095 mg/mL. At lower concentrations, eradication rates further declined to 47.85% at 0.012 mg/mL, 45.56% at 0.006 mg/mL, and 25.95% at 0.003 mg/mL. Overall, the EC/EtOAc extract demonstrated superior anti-adhesive and biofilm-eradication activities compared with the IC/EtOAc extract across all tested concentrations (Figure 9B).

##### Against *Staphylococcus aureus* (MRSA)

Adhesion assays against Methicillin-resistant *S. aureus* (MRSA) biofilm formation were performed using concentrations ranging from the MIC to 0.00075 mg/mL. The EC/EtOAc (3RR) extract exhibited strong anti-adhesive activity at higher concentrations, with 85.04% inhibition at 0.19 mg/mL and 75.07% at 0.095 mg/mL. A progressive decrease in activity was observed with dilution, reaching 66.76% at 0.024 mg/mL The lowest tested concentration (0.00075 mg/mL) exhibited only 5.43% inhibition. Statistical analysis demonstrated significant inhibition of MRSA biofilm formation at concentrations ≥ 0.024 mg/mL compared with the untreated positive control (*p* < 0.001).

The IC/EtOAc (3RR) extract demonstrated higher anti-adhesive activity against MRSA biofilms than the EC/EtOAc extract at equivalent concentrations. Maximum inhibition reached 87.18% at 0.39 mg/mL, followed by sustained inhibition of 80.75, 80.43, and 80.08% at 0.19, 0.095, and 0.048 mg/mL, respectively. Similar to the EC/EtOAc extract, inhibition dropped markedly at the lowest tested concentration (0.0015 mg/mL), reaching 5.43% (Figure 10A). The reproducibility of the assay was supported by low coefficients of variation at the most active concentrations (<10%).

**Figure 10:**
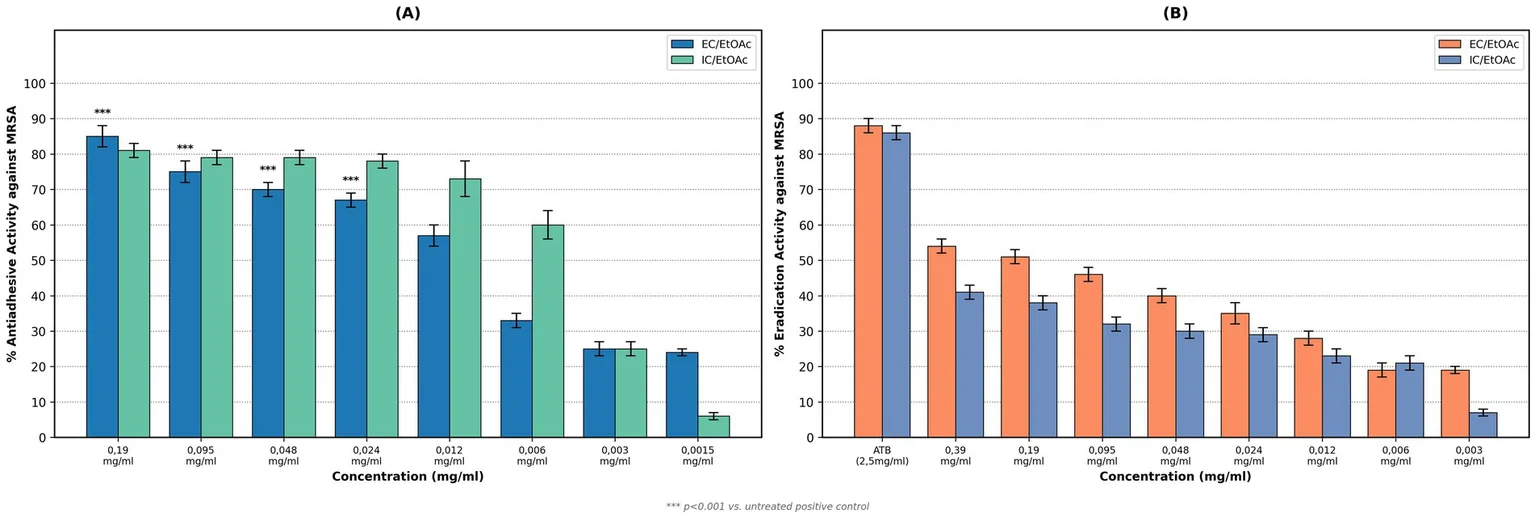
Antibiofilm activity against *S. aureus* (MRSA). Dose-dependent evaluation of antibiofilm activity of EC/EtOAc and IC/EtOAc extracts against methicillin-resistant *S. aureus* (MRSA). Concentrations ranged from 0.39 to 0.003 mg/mL. Anti-adhesive assays **(A)** measure inhibition of biofilm initiation, while eradication assays **(B)** evaluate disruption of established biofilms. ATB (2.5 mg/mL) was used as a positive antimicrobial control. Results are expressed as percentage inhibition relative to untreated controls (mean ± SD, *n* = 3).

The eradication activity of the EC/EtOAc (3RR) and IC/EtOAc (3RR) extracts against mature MRSA biofilms was evaluated over concentrations ranging from 0.78 mg/mL to 0.0015 mg/mL. The EC/EtOAc extract displayed the highest eradication activity at 0.39 mg/mL, with 54.85% inhibition, followed by 52.30% at 0.19 mg/mL Biofilm eradication progressively decreased at lower concentrations, reaching 28.58% at 0.012 mg/mL.

Comparatively, the IC/EtOAc extract showed lower eradication efficacy against mature MRSA biofilms. Maximum inhibition was observed at 0.78 mg/mL with 46.57% eradication. The activity gradually declined with decreasing concentrations, reaching 32.56% at 0.095 mg/mL, 30.60% at 0.048 mg/mL, and 29.39% at 0.024 mg/mL. At the lowest concentrations, inhibition rates further decreased to 24.39% at 0.012 mg/mL, 22.27% at 0.006 mg/mL, and 5.43% at 0.003 mg/m (Figure 10B). Overall, both extracts demonstrated stronger anti-adhesive effects than eradication activity against mature MRSA biofilms, with the IC/EtOAc extract being more effective during adhesion inhibition, whereas the EC/EtOAc extract showed comparatively better eradication capacity.

#### Confirmation of the antibiofilm activity by imaging

The efficacy of the EC/EtOAc (3RR) extract in inhibiting biofilm formation was demonstrated by optical microscopy, while its capacity to eradicate mature biofilms was confirmed by fluorescence microscopy.

The anti-adhesive and biofilm eradication effects of the EC/EtOAc (3RR) fungal extract were confirmed by optical and epifluorescence microscopy. Optical microscopy using crystal violet staining revealed clear differences between untreated (Figures 11A, 12A) and treated biofilms (Figures 11B–D, 12B–D) with a noticeable reduction in violet staining at 0.39 mg/mL, indicating decreased cellular adhesion and the formation of dispersed bacterial clusters. Similarly, fluorescence microscopy of mature biofilms stained with acridine orange showed that untreated biofilms formed dense, homogeneous layers, whereas treated biofilms exhibited disrupted architecture and fragmented colonies, with cells appearing as small aggregates. Overall, these observations confirm the significant anti-adhesive and biofilm-disrupting potential of the EC/EtOAc (3RR) extract against *S. epidermidis* and *S. aureus*.

**Figure 11:**
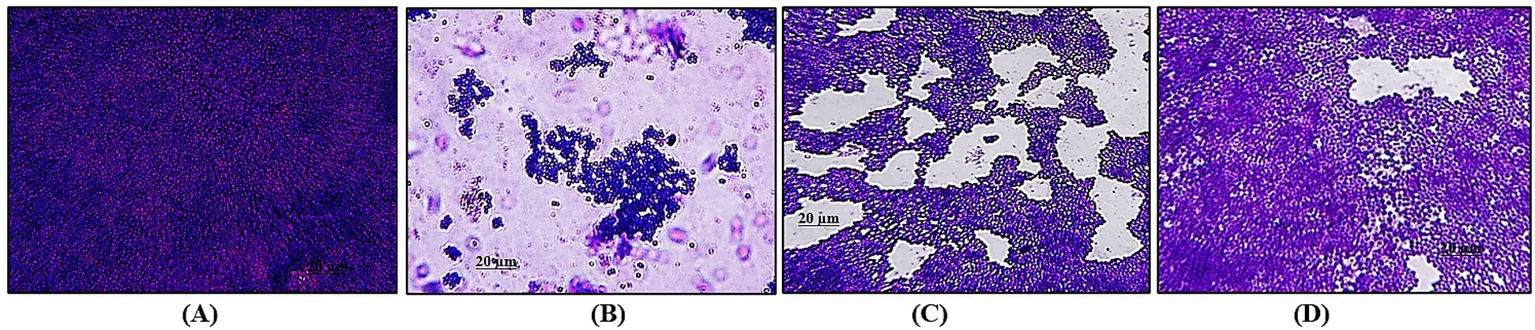
Optical microscopy of *S. epidermidis* biofilm inhibition. Optical microscopic evaluation of biofilm formation and inhibition by EC/EtOAc extract from *Fusarium* sp. 3RR against *S. epidermidis* (S61). **(A)** Untreated control exhibiting dense, structured biofilm architecture. **(B)** Treatment at 0.19 mg/mL (MIC) showing pronounced reduction in cell aggregation and extracellular matrix. **(C)** 0.024 mg/mL showing partial biofilm disruption. **(D)** 0.003 mg/mL showing minimal but detectable inhibition. Samples were stained with crystal violet for extracellular matrix visualization. Scale bars: 20 μm.

**Figure 12:**
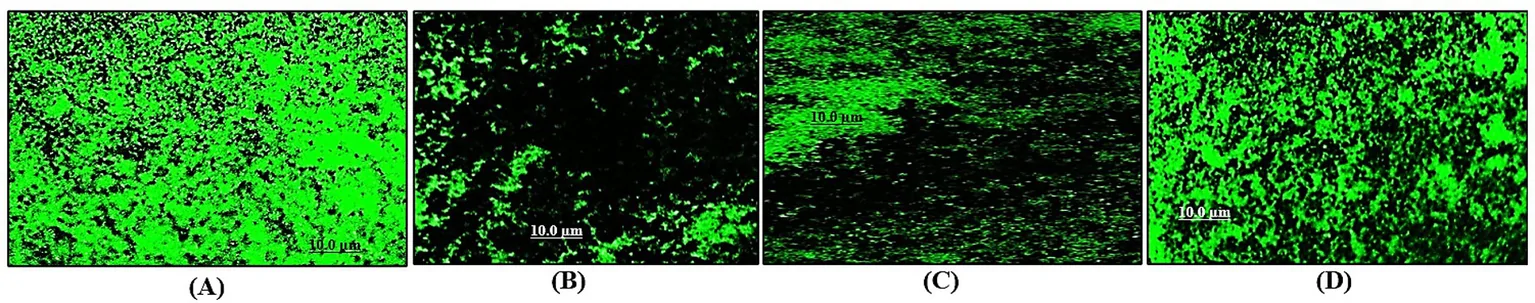
Fluorescence microscopy of MRSA biofilm eradication. Fluorescence microscopy analysis of biofilm viability and structural integrity of *S. aureus* (MRSA) following treatment with EC/EtOAc extract. **(A)** Untreated control displaying intact and metabolically active biofilm. **(B)** Treatment at 0.39 mg/mL (2 × MIC) showing strong reduction in viable biomass and matrix disruption. **(C)** 0.024 mg/mL demonstrating dose-dependent decrease in biofilm density. **(D)** 0.003 mg/mL showing partial survival of biofilm cells. Samples were stained with acridine orange for differentiation of live (green fluorescence) and metabolically compromised cells. Scale bars: 10 μm.

#### GC-MS analysis of the EC/EtOAc extract from *Fusarium* sp. (3RR)

The extracellular fungal extract obtained by ethyl acetate extraction (EC/EtOAc) exhibited promising antibacterial and antibiofilm activities against the tested strains. Optical and fluorescence microscopy analyses, evaluating biofilm formation inhibition and mature biofilm eradication, confirmed these activities with significant results. Gas chromatography coupled to mass spectrometry (GC-MS) analysis of the crude extract identified diverse chemical groups with important biological activities (Supplementary Figure S10).

GC-MS analysis of the EC/EtOAc (3RR) fungal extract revealed its major chemical constituents (Table 3), among which 2,4-di-tert-butylphenol (2,4-DTBP) was the most abundant, accounting for 16.72% of the total composition.

**Table 3:** Compounds identified by gas chromatography–mass spectrometry (GC-MS) analysis in the ethyl acetate extracellular extract of *Fusarium* sp. 3RR.

S#	Compound	RT	Area %	Formula	Class	Metabolites or Precursor
1	2,4-Di-tert-butylphenol	5.506	16.72	C₁₄H₂₂O	Phenolic compound	Tyrosine → phenylpropanoid pathway (Ali et al., 2018)
2	7-Hexadecene, (Z)-	6.043	5.25	C₁₆H₃₂	Alkene	Palmitic acid → decarboxylation (Perera et al., 2010)
3	Methanone, (1-hydroxycyclohexyl) phenyl-	7.68	1.18	C₁₃H₁₆O₂	Aromatic ketone	Phenylalanine → aromatic catabolism
4	E-15-Heptadecenal	8.529	9.27	C₁₉H₃₈	Unsaturated aldehyde	Stearic acid → β-oxidation/peroxidation (Perera et al., 2010)
5	1-Nonadecene	13.448	10.1	C₁₉H₃₈	Long-chain alkene	Arachidic acid → decarboxylation
6	Benzenepropanoic acid ester	14.123	1.12	C₁₉H₃₀O₃	Phenolic ester	Cinnamic acid → esterification
7	Cyclotetradecane	17.399	1.62	C₁₄H₂₈	Cyclic alkane	Myristic acid → cyclization
8	1-Docosene	22.713	6.73	C₂₂H₄₄	Very long-chain alkene	Behenic acid → decarboxylation (Perera et al., 2010)
9	1-Dodecanol, 3,7,11-trimethyl-	23.481	2.24	C₁₅H₃₂O	Branched fatty alcohol	Farnesol → reduction (isoprenoid pathway) (Clomburg et al., 2019)
10	8-Hydroxyisotrichodermin	26.63	7.79	C₁₇H₂₄O₅	Trichothecene mycotoxin	Mevalonate → farnesyl-PP → trichothecene cluster (McCormick et al., 2011; Gao et al., 2025)
11	Nonacos-1-ene	29.594	4.67	C₂₉H₅₈	Very long-chain alkene	Melissic acid → decarboxylation (Perera et al., 2010)
12	Phthalic acid, decyl octyl ester	33.525	3.53	C₂₆H₄₂O₄	Phthalate ester	–
13	10-Heneicosene	34.555	2.76	C₂₁H₄₂	Long-chain alkene	Behenic acid → decarboxylation (Perera et al., 2010)
14	Oleic Acid	37.457	10.14	C₁₈H₃₄O₂	Monounsaturated fatty acid	Acetyl-CoA → fatty acid synthase → Δ9-desaturase (Hu et al., 2023)

RT, retention time. The table lists the serial number (S#), compound name, retention time (RT), relative peak area percentage (Area %), molecular formula (Formula), chemical class (Class), and whether the compound is a metabolite or a biosynthetic precursor.

## Discussion

The increasing prevalence of antimicrobial resistance and biofilm-associated infections underscores the urgent need for novel bioactive compounds. Endophytic fungi, with their remarkable metabolic versatility and adaptation to diverse ecological niches, represent a promising, yet underexplored, reservoir of such molecules (Manganyi and Ateba, 2020; Silva et al., 2022; Wen et al., 2022; Nazir et al., 2024; Kubayi et al., 2025). This study employed an integrated genomics-metabolomics approach to explore the biosynthetic potential of *Fusarium oxysporum* strain 3RR and to identify possible associations between its genomic architecture, enzymatic repertoire, and the observed antibiofilm and antibacterial activities. Our findings offer preliminary evidence consistent with how this endophytic fungus may express its genetic potential as a bioactive metabolite profile.

Notably, this work extends beyond genome-guided natural product discovery by combining a high-quality hybrid genome assembly, BGC prediction, GC-MS metabolite profiling, and quantitative antibiofilm assays in a single plant-endophytic *F. oxysporum* isolate. In doing so, it provides an integrated ecological and functional framework that is still rarely available for plant-associated non-pathogenic members of the *Fusarium oxysporum* species complex. This contrasts with prior work that has largely focused on pathogenic *F. oxysporum* genomes or on a small number of non-pathogenic reference strains such as Fo47 (Wang et al., 2020b; Constantin et al., 2021), while comparative studies have shown that endophytic and pathogenic strains differ in accessory genome features and effector repertoires (He et al., 2023). Importantly, the novelty of this work lies not in the detection of known compounds alone, but in placing these metabolites within their genomic and ecological context, extending earlier reports of 2,4-DTBP from endophytic *F. oxysporum* that lacked genomic characterization of the producing strain (Sundar and Arunachalam, 2025).

The whole-genome sequencing and hybrid assembly of *F. oxysporum* 3RR yielded a high-quality genome (41.14 Mb, 12 contigs, N50 of 3.52 Mb) with a GC content of 48.18%, consistent with typical fungal genomes. This assembly allowed for detailed functional annotation and biosynthetic gene cluster (BGC) prediction. The annotation pipeline identified a significant number of genes involved in metabolic and regulatory processes, with a notable proportion dedicated to secondary metabolism, highlighting the organism’s inherent capacity for producing diverse natural products.

A key aspect of our functional analysis was the identification of carbohydrate-active enzymes (CAZy). Glycoside hydrolases (GH) were particularly abundant, with prominent GH18 (chitinases) and GH3 (exoglycosidases) subfamilies. These enzymes are crucial for the degradation of plant cell walls and fungal chitin, suggesting that *F. oxysporum* 3RR efficiently acquires carbon sources from its environment, including the plant host (as an endophyte) or other organic matter (as a saprophyte). This aligns with the understanding of Fusarium species as versatile decomposers and colonizers (Morelli et al., 2020). The presence of these degradative enzymes acts as the initial processing stage, providing the necessary carbon substrate (glucose and monosaccharides) to fuel the organism’s central metabolism (Catharina and Carels, 2018; Roy et al., 2020; Martínez-Soto et al., 2023).

The antiSMASH analysis revealed 36 putative BGC in *F. oxysporum* 3RR, predominantly terpenes, non-ribosomal peptide synthetases (NRPS), and polyketide synthases (PKS), along with isocyanide and hybrid clusters. This diversity highlights the strong biosynthetic potential of the strain, with several clusters confidently associated with known metabolites such as clavariic acid, koraiol, α-acorenol, aurofusarin, oxyjavanin, ilicicolin H, and T-2 toxin. Terpenes like α-acorenol may play roles in host interaction and specificity (Kharte et al., 2025), while PKS/NRPS-derived compounds such as aurofusarin contribute to ecological fitness, including UV protection (Hoogendoorn et al., 2018).

The following mechanistic interpretations are hypothesis-driven and derive from genomic architecture rather than direct experimental evidence. The genomic distribution of these clusters suggests a functional organization and potential for co-regulation or substrate channeling. In fact, Contig 8’s 6 clusters (terpene-NRPS hybrids + PKS) may represent a hotspot of biosynthetic innovation, potentially analogous to the enrichment of biosynthetic gene clusters observed on supernumerary chromosomes in *Fusarium* species. This clustering could facilitate coordinated expression and functional redundancy, thereby maintain core metabolic functions while allowing diversification of secondary metabolites. This genomic arrangement may promote evolutionary flexibility. It may also support ecological adaptation and host specificity. In addition, it could contribute to the production of novel bioactive compounds (Li et al., 2014; Hoogendoorn et al., 2018; Kharte et al., 2025).

The identification of PHI-base homologs in *Fusarium oxysporum* 3RR, characterized by substantial sequence identity and coverage, reveals a predominance of reduced virulence (50.6%) and unaffected pathogenicity (30.9%) phenotypes. This distribution is consistent with an endophytic lifestyle, where attenuation of virulence is often associated with the establishment of mutualistic interactions. In comparison to pathogenic strains such as *F. oxysporum* f. sp. *lentis*, which exhibit a higher number of PHI-base hits and a stronger association with moderate virulence traits (Kharte et al., 2025), the presence of 186 genes displaying dual phenotypes in 3RR suggests context-dependent functional plasticity. This pleiotropic behavior may reflect adaptive flexibility exceeding that observed in other *Fusarium* species.

Comparative phylogenomic analyses further support a balance between conservation and adaptation. PHAST results indicate a high level of sequence conservation, with a large proportion of phylogenetically conserved sites, consistent with the genomic collinearity reported within the *F. fujikuroi* species complex (Wiemann et al., 2013). Nevertheless, the presence of accelerated regions highlights ongoing evolutionary dynamics that may underpin ecological adaptation (Rau et al., 2024). Notably, the relatively limited gene duplication compared to pathogenic strains indicates a restrained genome expansion strategy, potentially favoring functional stability in endophytic contexts (Hill et al., 2022; Kharte et al., 2025).

Functional characterization of species-specific orthologs underscores their relevance in regulatory and metabolic processes. Importantly, the high proportion of uncharacterized proteins points to unexplored functional diversity, potentially linked to endophytic adaptation and ecological specialization (Wojtasik et al., 2024). Collectively, these findings highlight the distinct genomic features of strain 3RR, which appear to support a shift from pathogenicity toward a more beneficial or neutral interaction with the host (Hill et al., 2022).

The GC-MS analysis of the extracellular ethyl acetate extract provided experimental validation of the predicted biosynthetic potential, identifying 14 major compounds with putative associations to predicted BGCs, based on bioinformatic correlation rather than functional confirmation.

The most abundant compound was 2,4-di-tert-butylphenol (2,4-DTBP), representing 7.24% of detected metabolites, which is predicted by PKS clusters (Regions 3.1, hybrid PKS-NRPS architecture). 2,4-DTBP has been reported as an antimicrobial compound with membrane-disruptive activity in bacteria and fungi (Fan et al., 2023; Yang et al., 2024; Sundar and Arunachalam, 2025), consistent with and potentially contributing to the antibacterial potency of the EtOAc extract, though direct attribution to this single compound cannot be established from crude extract data alone.

This raises the hypothesis of a two-step process in which PKS enzymes generate precursor compounds, and CE10 esterases may potentially contribute to the liberation of free phenolic derivatives, an interpretation grounded in bioinformatic annotation and analogy to related fungal systems (Zhang et al., 2022a), but not supported by direct enzymatic evidence in the present study.

This proposed post-biosynthetic modification by CAZymes such as CE10 may represent a control point for the organism to regulate the activity and availability of its secondary metabolites (Roy et al., 2020; Cawood and Ton, 2025) however, no enzymatic assays were conducted to confirm CE10 activity or its direct involvement in biofilm-associated outcomes.

Another major component detected by GC-MS was oleic acid along with numerous degradation products such as alkenes and aldehydes, collectively comprising 35–40% of the profile. While oleic acid is a primary metabolite, its presence in the extracellular extract and subsequent degradation products could be directly linked to the activity of CE10, functioning as a lipase. CE10 releases free fatty acids from lipid esters, which then undergo β-oxidation (Novak et al., 2015). This suggests that *F. oxysporum* 3RR actively mobilizes and metabolizes lipids, possibly for energy or to generate signaling molecules. Also, CE10 may represent a putative metabolic interface between primary lipid metabolism and the external environment, though direct enzymatic validation remains to be established. This finding is consistent with oleic acid’s known role as an effective antibiofilm agent (Nazzaro et al., 2026) which supports the observed antibiofilm activity of the fungal extracts.

8-hydroxyisotrichodermin, a trichothecene sesquiterpenoid (7.79% of metabolites), is predicted by terpene synthase clusters (Regions 2.1, 10.3, 12.4, with CYP450 modifications). Trichothecenes are known biofilm inhibitors via exopolysaccharide disruption and quorum-sensing interference (Zhang J. et al., 2022). For instance, trichothecenes, are also known virulence factors in *Fusarium* species, enabling plant pathogenicity by inhibiting protein synthesis and causing cell death (Lee et al., 2020). The high abundance of 8-hydroxyisotrichodermin is particularly significant and is possibly linked to the activity of auxiliary activity (AA9) enzymes, specifically lytic polysaccharide monooxygenases (Wang et al., 2020a). AA9 enzymes generate reactive oxygen species during polysaccharide degradation, which acts as a stress signal, upregulating CYP450 monooxygenases involved in the oxidative biosynthesis of trichothecenes (Hoogendoorn et al., 2018). This highlights a stress-induced mechanism where the organism responds to host polysaccharides by producing potent mycotoxins, thereby enhancing its virulence (Ponts, 2015; Rauwane et al., 2020). This regulatory pathway illustrates how environmental signals can trigger fungal chemical defense mechanisms. Farnesol, a precursor in trichothecene biosynthesis, may act both as a quorum-sensing modulator and as an intermediate of the mevalonate pathway involved in trichothecene production. These roles may contribute to the observed biofilm eradication activity (Cardoza et al., 2022).

These compound-to-BGC-to-bioactivity integration suggests that the identified metabolites may be functionally relevant contributors to the strain’s antimicrobial phenotype (Zhu et al., 2026). The metabolomic data are consistent with the bioinformatics predictions and provide a hypothesis-generating framework for observed bioactivities, pending experimental validation (Supplementary Figure S11).

The observed antibiofilm and antibacterial activities of the *F. oxysporum* 3RR extracts corroborate the predicted metabolic profile. The EC/EtOAc extract showed significant antibacterial activity against *S. aureus*, *S. epidermidis*. These results are consistent with the known antimicrobial properties of phenolic compounds like 2,4-DTBP and fatty acids such as oleic acid, both abundantly detected in the extract (Viszwapriya et al., 2016; Sundar and Arunachalam, 2025). Furthermore, the anti-adhesive and eradication activities against *S. epidermidis* and *S. aureus* biofilms were remarkably high, which was qualitatively supported by optical and fluorescence microscopy. It is acknowledged that these images provide illustrative, rather than quantitative, evidence of biofilm disruption; the crystal violet assay itself is a semi-quantitative method reflecting total biomass and does not distinguish between viable and non-viable cells.

The ability of 2,4-DTBP to modulate extracellular polymeric substances (EPS) and enhance antimicrobial penetration into biofilms provides a strong mechanistic basis for these observed antibiofilm effects. Similarly, alkenes like E-15-heptadecenal have demonstrated antibacterial activity by inducing cellular deformations. Multiple studies have demonstrated that 2,4-DTBP disrupts EPS by reducing protein/polysaccharide content, increasing matrix hydration, and decreasing crystallite size/crystallinity, thereby facilitating antimicrobial penetration into biofilms (Padmavathi et al., 2015; Viszwapriya et al., 2016; Zhao et al., 2020).

### Limitations

By combining whole-genome sequencing, BGC prediction, and metabolomic profiling with preliminary bioactivity assays, this study establishes an integrative genomic–functional framework that positions *F. oxysporum* 3RR as a biologically relevant source of bioactive compounds. These findings contribute to the growing paradigm of endophytic fungi as biofilm inhibitors (Zhu et al., 2026), situating this strain within a broader landscape of plant-associated microbes, including dandelion endophytes (Liu et al., 2025) and marine-derived strains (Jamal et al., 2025), in which BGC-derived phenols and fatty acids disrupt EPS matrices and interfere with quorum sensing. In doing so, this work challenges the reductive view of *F. oxysporum* as solely pathogenic and advances the “silent genome” hypothesis by demonstrating that endophytic isolates harbor cryptic biosynthetic pathways with both ecological and therapeutic relevance. The identification and preliminary functional correlation of 36 BGCs with observed antibiofilm and antibacterial activities provides a coherent, evidence-consistent foundation for repositioning this species as a candidate pharmaceutical resource.

When interpreting these findings, several methodological boundaries should be acknowledged. Despite a high-quality chromosome-scale assembly, residual genomic fragmentation may have partially obscured minor or overlapping BGCs. Furthermore, mechanistic interpretations — including the proposed roles of CE10 carbohydrate esterases in post-biosynthetic metabolite modification and the contribution of CAZy enzyme activity to the observed antibiofilm phenotype—remain putative at this stage. These interpretations are grounded in bioinformatic predictions and are consistent with published biochemical data from related fungal systems; however, they have not yet been directly validated through enzymatic assays, transcriptomic profiling, or gene knockout experiments. These represent natural next steps rather than fundamental weaknesses, and the integrative framework established here is precisely designed to guide and prioritize such future investigations.

### Future directions

Future work should focus on the validation and molecular characterization of the identified metabolites using high-resolution analytical techniques, such as GC-MS/MS, to improve the confidence of compound identification. In particular, key metabolites including 2,4-DTBP, oleic acid, and 8-hydroxyisotrichodermin should be further investigated to confirm their identities and evaluate their individual and synergistic contributions to the observed antibiofilm activity. Their mechanisms of action can then be investigated against clinical MDR *staphylococci* and *Candida albicans*, including quorum sensing interference and effects on the EPS matrix. In parallel, integrating RNA-seq under biofilm and stress conditions would help link BGC expression to metabolite production, with functional validation through targeted gene knockouts.

## Conclusion

In conclusion, this integrated systems biology analysis of *Fusarium oxysporum* 3RR reveals a sophisticated metabolic architecture driven by a rich repertoire of CAZy enzymes and secondary metabolite BGCs. The synergy between carbohydrate degradation stress signaling, primary metabolism, and diverse secondary metabolite biosynthesis (PKS, NRPS, Terpenes, Isocyanides) culminates in the production of a bioactive metabolite profile. The resulting GC-MS profile is consistent with and suggestive of a molecular basis for the observed biological activities. This study provides evidence consistent with how the genomic potential of *F. oxysporum* 3RR may be expressed as a bioactive metabolite profile, positioning this endophytic fungus as a candidate source for novel therapeutic agents against microbial biofilms.

## Data Availability

The datasets presented in this study can be found in online repositories. The names of the repository/repositories and accession number(s) can be found in the article/Supplementary material.

## Footnotes

1
http://www.NCBI.nim.nih.gov/

2
https://cagecat.bioinformatics.nl/
