# Supplementary material for: The endophyte Fusarium oxysporum 3RR genome analysis identifies bioactive compounds targeting biofilm formation
Source: Front Microbiol. 2026 Sep 21;17:1841716. doi: 10.3389/fmicb.2026.1841716 (PMC13635361; doi:10.3389/fmicb.2026.1841716)
Supplement: Supplementary file 1 [file Table_1.DOCX]

Supplementary Material

# 1 Supplementary Figures and Tables

## Supplementary Table S1: media composition

| Media | Full name | Composition |
| --- | --- | --- |
| PDB | Potato Dextrose Broth | dehydrated potato 200 g/L, 20 g dextrose. |
| PDA | Potato Dextrose Agar | dehydrated potato 200 g/L, 20 g/L dextrose, agar 18g/L |
| MHA | Mueller-Hinton Agar | 17.5g acid hydrolysate of casein, 2.0g beef extract, 1.5g starch, and 17.0g agar |
| MHB | Mueller-Hinton Broth | Beef Infusion Solids (2.0 -3.0 G/L), acid hydrolysate (17.5 g/L, and Soluble Starch (1.5g/L) |
| TSB | Tryptic Soy Broth | Tryptone 17.0 g, Soya peptone 3g, NaCl 3g, K2HPO4 2.5g, glucose 2.5g |

**Supplementary Table S2**: Macroscopic and Microscopic Characterization of Fusarium sp. (3RR)

| **Macroscopic aspect** | | **Microscopic aspect** | | **Characteristics** |
| --- | --- | --- | --- | --- |
| ***Fusarium.*sp. (3RR)** | | | | |
|  |  |   **40X** |   **100X** | *Obverse: Fluffy appearance with a cottony texture and white coloration, exhibiting a soft and silky aspect reminiscent of wool or cotton.  *Reverse: Reddish to dark brown coloration.  *Microscopic observation: Visible structures consist of long, thin, and segmented hyphae (filaments) displaying regular septa along each filament. The hyphae appear smooth and septate, with minimal or no branching. |

Supplementary Table S3: Quality control, trimming, filtering, and merging of sequencing reads for Fusarium sp.

| Step | Total Sequences | Total Bases | Sequence Length (bp) | %GC |
| --- | --- | --- | --- | --- |
| Raw reads (QC) | 10 637 990 | 1.5 Gbp | 35-149 | 47 |
| After trimming & filtering | 6 769 045 | 990.8 Mbp | 53-149 | 47 |
| After merging | 2 463 817 | 528 Mbp | 73-267 | 47 |

Supplementary Table S4: Distribution of InterProScan Annotations Across Predicted Proteins

| Functional annotation | Count |
| --- | --- |
| MobiDBLite | 33319 |
| Pfam | 14333 |
| Gene3D | 12951 |
| SUPERFAMILY | 10330 |
| PANTHER | 9204 |
| PIRSR | 7397 |
| PRINTS | 6359 |
| SMART | 5656 |
| ProSiteProfiles | 5541 |
| CDD | 4819 |
| FunFam | 3980 |
| Coils | 3161 |
| ProSitePatterns | 2457 |
| NCBIfam | 1172 |
| PIRSF | 792 |
| Hamap | 351 |
| SFLD | 202 |

**Supplementary Table S5**: High Confidence Secondary Metabolite Biosynthetic Gene Clusters Identified in Fusarium oxysporum by antiSMASH Analysis

| Region ID | Location | Cluster Type | Known Compound | Pathway Classification | Size (bp) |
| --- | --- | --- | --- | --- | --- |
| 1.3 | 501,875-565,723 | NRPS-like | Choline | NRPS:Type I | 63,848 |
| 2.1 | 571,218-603,623 | Terpene | Clavaric acid | Sesquiterpene | 32,405 |
| 3.1 | 488,652-555,776 | Type I PKS | Oxyjavanacin | PKS | 67,124 |
| 9.1 | 177,972-292,598 | Hybrid (T1PKS/NRPS-like/Isocyanide) | Aurofusarin | Polyketide | 114,626 |
| 10.3 | 1,597,344-1,629,006 | Terpene | Koraiole | Sesquiterpene | 31,662 |
| 12.4 | 1,540,906-1,572,187 | Terpene | α-Acorenol | Sesquiterpene | 31,281 |

**Supplementary Table S6**: Production and liquid/liquid extraction of secondary metabolites produced by 3RR fungal endophyte

| **Submerged culture** | **Mycelium biomass** | **Intracellular extracts** | **Yield (mg/g of biomass)** | **Culture filtrate** | **Extracellular extracts** | **Yield**  **(mg ou g/ L**  **of culture)** |
| --- | --- | --- | --- | --- | --- | --- |
|  | | | | | | |
| ***Fusarium* sp. (3RR)** | | | | | | |
|  |  | **MeOH EtOAc** | **EtOAc : 25±3.2 mg/g**  **MeOH : 35.7±1.2 mg/g** |  | **MeOH EtOAc** | **EtOAc: 107.5±6.2 mg/ml**  **MeOH : 2.14±0.12 mg/ml** |

Extracellular fraction extracted with methanol from strain 3RR, IC/MeOH (3RR): Intracellular fraction extracted with methanol from strain 3RR

Supplementary Table S7: Antibacterial activity of EC and IC using the agar diffusion method

|  | **Diameter of inhibition (mm)** | | | | |
| --- | --- | --- | --- | --- | --- |
| **Fungal extract** | ***Staphylococcus aureus*** | ***Staphylococcus epidermidis*** | ***Pseudomonas aeruginosa*** | ***Escherichia coli*** | ***Serratia marcescens*** |
| **EC/EtOAc (3RR)** | **[12-14]** | **[32-34]** | **0** | **[29-31]** | **[29-31]** |
| **IC/EtOAc (3RR)** | **[10-12]** | **0** | **0** | **0** | **0** |
| **EC/MeOH (3RR)** | **0** | **0** | **0** | **0** | **0** |
| **IC/MeOH (3RR)** | **0** | **0** | **0** | **0** | **0** |
| **C+ (ATB 2,5mg/ml)** | **[32-34]** | **[31-32]** | **[18-21]** | **[34-35]** | **[31-35]** |
| **C- (DMSO)** | **0** | **0** | **0** | **0** | **0** |

mm: millimeter, EC/EtOAc (3RR): Extracellular fraction extracted with ethyl acetate from strain 3RR, IC/EtOAc (3RR): Intracellular fraction extracted with ethyl acetate from strain 3RR, EC/MeOH (3RR): Extracellular fraction extracted with methanol from strain 3RR, IC/MeOH (3RR): Intracellular fraction extracted with methanol from strain 3RR, C+: positive control, C-: negative control

Supplementary Figures

Supplementary Figure S1: Schematic Summary of Liquid-Liquid Production and Extraction of Fungal Metabolic Extracts
(Created using <https://www.canva.com/>, professional version, invoice n° 04649 -15652816)

**Supplementary Figure S2**: Workflow for MIC Determination Using a 96-Well Plate MTT-Based Assay

(Created using <https://www.canva.com/>, professional version, invoice n° 04649 -15652816). Schematic representation of the experimental workflow for determining the Minimum Inhibitory Concentration (MIC) using a microdilution method combined with an MTT viability assay. (1) Preparation of a 96-well plate with test extracts, antibiotic control, and serial dilutions. (2) Inoculation with bacterial suspension in Mueller–Hinton Broth (MHB) followed by incubation at 37°C for 18–24 hours. (3) Addition of MTT reagent to assess bacterial metabolic activity, with viable cells producing a color change. (4) Determination of MIC as the lowest extract concentration that inhibits visible bacterial growth, indicated by lack of color conversion.

Supplementary Figure S3. Percentage of nanopore reads above Q-Score cutoffs (raw vs processed)

Supplementary Figure S4. Estimated genome size across K-mer sizes from K-mer analysis

Supplementary Figure S5. Genome-wide distribution of repetitive elements identified by RepeatMasker

**Supplementary Figure S6**: Top 20 Pfam domains by occurrence with functional annotation

Supplementary Figure S7: Frequency of Enzyme Commission (EC) Numbers

**Supplementary Figure S8**: Protein domain architecture and functional annotation of 106 species-specific orthologs based on InterPro analysis; (A) Top 15 Protein Domains and Families Ranked by Frequency (B) Distribution of Protein Domains Across Ten Functional Categories

**Supplementary Figure S9**. Yield of extraction of secondary metabolites produced by the fungi 3RR

Supplementary Figure S10: GC-MS chromatogram obtained with the ethyl acetate extract from the supernatant of RR endophytic fungal strain

**Supplementary Figure S11: Integrative Model of Biosynthetic Gene Cluster (BGC) Predictions** Linked to Identified Secondary Metabolites and Bioactivities in Fusarium oxysporum 3RR Metabolic integration diagram showing the predicted relationship between identified biosynthetic gene clusters (antiSMASH predictions, shown in colored boxes) and secondary metabolites detected by GC-MS analysis (metabolite names in italics). The diagram maps the primary metabolic hub (central carbon metabolism, top) branching into three major secondary metabolite biosynthetic pathways: (1) Polyketide synthase (PKS) pathway (left, green boxes) generating lipophilic polyketides including 2,4-DTBP and fatty acid derivatives (oleic acid, linoleic acid); (2) Terpene/isoprenoid pathway (right, blue boxes) producing sesquiterpenoids and volatile organic compounds including 8-hydroxyisotrichodermin and geranyl compounds; and (3) Hybrid pathways involving fatty acid oxidation and post-translational modifications (middle). Each pathway is annotated with key enzymatic domains (PKS, PCP, KS, AT, KR) identified from antiSMASH analysis. Bottom connections (red boxes) show how identified metabolite classes correlate with observed bioactivities: lipophilic compounds (2,4-DTBP, oleic acid) → antibacterial activity via cell membrane disruption; terpenoids (trichothecenes, geranyl derivatives) → biofilm eradication and anti-adhesion; and polar metabolites (not shown in left/right pathways) → complementary biofilm inhibition via alternative mechanisms. This integration of genomic predictions (BGCs, antiSMASH), metabolomic analysis (GC-MS identification), and bioactivity data demonstrates the functional relevance of predicted biosynthetic pathways and validates the endophytic strain's capacity to produce multiple classes of bioactive secondary metabolites.
